# Transforming growth factor-β1 decreases erythropoietin production through repressing hypoxia-inducible factor 2α in erythropoietin-producing cells

**DOI:** 10.1186/s12929-021-00770-2

**Published:** 2021-11-02

**Authors:** Hong-Mou Shih, Szu-Yu Pan, Chih-Jen Wu, Yu-Hsiang Chou, Chun-Yuan Chen, Fan-Chi Chang, Yi-Ting Chen, Wen-Chih Chiang, Hsing-Chen Tsai, Yung-Ming Chen, Shuei-Liong Lin

**Affiliations:** 1 Graduate Institute of Physiology, College of Medicine, National Taiwan University, No. 1, Jen-Ai Road Section 1, Taipei, 100 Taiwan; 2 Division of Nephrology, Department of Internal Medicine, Mackay Memorial Hospital, Taipei, Taiwan; 3 Department of Integrated Diagnostics and Therapeutics, National Taiwan University Hospital, Taipei, Taiwan; 4 Renal Division, Department of Internal Medicine, National Taiwan University Hospital, Taipei, Taiwan; 5 Division of Nephrology, Department of Internal Medicine, Far Eastern Memorial Hospital, New Taipei City, Taiwan; 6 Department of Medicine, Mackay Medical College, Taipei, Taiwan; 7 Department of Pharmacology, Graduate Institute of Medical Sciences, College of Medicine, Taipei Medical University, Taipei, Taiwan; 8 Department of Internal Medicine, National Taiwan University Hospital Jin-Shan Branch, New Taipei City, Taiwan; 9 School of Medicine, College of Medicine, National Taiwan University, Taipei, Taiwan; 10 Division of Chest Medicine, Department of Internal Medicine, National Taiwan University Hospital, Taipei, Taiwan; 11 Graduate Institute of Toxicology, College of Medicine, National Taiwan University, Taipei, Taiwan; 12 Research Center for Developmental Biology and Regenerative Medicine, National Taiwan University, Taipei, Taiwan

**Keywords:** Erythropoietin, Hypoxia-inducible factor 2α, Pericyte, Transforming growth factor-β1

## Abstract

**Background:**

Renal erythropoietin (EPO)-producing (REP) cells produce EPO through hypoxia-inducible factor (HIF) 2α-activated gene transcription. Insufficient EPO production leads to anemia in patients with chronic kidney disease. Although recombinant EPO is effective to improve anemia, no reliable REP cell lines limit further progress of research and development of novel treatment.

**Methods:**

We screened *Epo* mRNA expression in mouse fibroblast cell lines. Small interfering RNA specific for HIF1α or HIF2α was transfected to study *Epo* expression in C3H10T1/2 cells. The effect of transforming growth factor-β1 (TGF-β1) on HIF-EPO axis was studied in C3H10T1/2 cells and mice.

**Results:**

Similar to mouse REP pericytes, C3H10T1/2 cells differentiated to α-smooth muscle actin^+^ myofibroblasts after exposure to TGF-β1. Specific HIF knockdown demonstrated the role of HIF2α in hypoxia-induced *Epo* expression. Sustained TGF-β1 exposure increased neither DNA methyltransferase nor methylation of *Epas1* and *Epo* genes. However, TGF-β1 repressed HIF2α-encoding *Epas1* promptly through activating activin receptor-like kinase-5 (ALK5), thereby decreasing *Epo* induction by hypoxia and prolyl hydroxylase domain inhibitor roxadustat. In mice with pro-fibrotic injury induced by ureteral obstruction, upregulation of *Tgfb1* was accompanied with downregulation of *Epas1* and *Epo* in injured kidneys and myofibroblasts, which were reversed by ALK5 inhibitor SB431542.

**Conclusion:**

C3H10T1/2 cells possessed the property of HIF2α-dependent *Epo* expression in REP pericytes. TGF-β1 induced not only the transition to myofibroblasts but also a repressive effect on *Epas1*-*Epo* axis in C3H10T1/2 cells. The repressive effect of TGF-β1 on *Epas1*-*Epo* axis was confirmed in REP pericytes in vivo. Inhibition of TGF-β1-ALK5 signaling might provide a novel treatment to rescue EPO expression in REP pericytes of injured kidney.

**Supplementary Information:**

The online version contains supplementary material available at 10.1186/s12929-021-00770-2.

## Background

Renal erythropoietin (EPO)-producing (REP) cells are interstitial platelet-derived growth factor receptor-β (PDGFRβ)^+^ pericytes, also known as perivascular fibroblasts [1–6]. In addition to produce EPO under conditions of anemia and hypoxia, pericytes produce growth factors to promote angiogenesis or microvascular stability [6–9]. In injured kidneys, however, pericytes are the precursors of myofibroblasts [3, 9–13]. Transforming growth factor (TGF)-β1, one of pro-fibrotic cytokines involved in the pathogenesis of renal fibrosis, can upregulate DNA methyltransferase (DNMT) which increases methylation of *Rasal1* and *Ybx2* genes in pericytes, thereby activating cell proliferation and α-smooth muscle actin (α-SMA) expression, respectively [9, 14]. Concomitant hypermethylation in the 5′-enhancer and promoter of *Epo* gene results in the repression of *Epo* in myofibroblasts and anemia in chronic kidney disease (CKD) [3]. In addition to *Epo* methylation, a variety of pathogenetic factors in CKD including inflammation, endoplasmic reticulum stress, oxidative stress and uremic toxin have been shown to reduce EPO production in the cell or animal models [1, 2, 15–20].

The kidney and liver produce about 90% and 10% of EPO in the adult, respectively, while the liver is the main source of EPO in the fetus [21–23]. Hence it is reasonable to use hepatic cell lines in the study of *Epo* gene regulation [16, 18, 24–26]. Decreased tissue oxygen tension appears to be the major factor triggering EPO production through hypoxia-inducible factor (HIF)-activated transcription [27, 28]. While the hypoxia response element (HRE)^+^ 3′-enhancer of *Epo* gene is liver specific [26, 29], Storti et al. report a functional HIF2α-dependent HRE in the distal 5′-enhancer located at kidney-inducible element (KIE) [30]. A number of attempts to generate REP cell lines have been reported [31–33]. In fibroblast-like 4E cells isolated from the adult mouse kidney, the subset of CD73^+^ cells are capable of expressing *Epo* under hypoxia [31]. REP cells isolated from severe neonatal anemic mice (*Epo*^*GFP/Δ3′E*^) provide the evidence of HIF in the regulation of renal EPO production [32]. Renal CD133^+^/CD73^+^ mesenchymal progenitor cells, isolated from the inner medulla of human kidneys, produce EPO under hypoxia through HIF2α [33]. We also provide evidence that primary Col1a1-GFP^+^ pericytes isolated from *Col1a1-GFP*^*Tg*^ mice are capable of producing EPO under hypoxia [3]. However, Col1a1-GFP^+^ pericytes differentiate to myofibroblasts after passages and decrease *Epo* expression due to hypermethylation in the promoter and 5′-distal enhancer of *Epo* gene [3]. Hence, no success has been achieved in establishing reliable REP cell lines with capability of regulated EPO expression until recently [19, 20]. Fibroblastoid atypical interstitial kidney (FAIK) cells are obtained by isolation of primary REP cells from *Epo-CreERT2*^*Tg*^;*Rosa26*^*fstdTomato/*+^ mice and then immortalized with SV40 large T antigen [19]. FAIK cells maintain hypoxia-induced EPO expression, even at rather low levels, for at least 30 passages. Interestingly, demethylating agent 5-azacytidine can enhance both basal and hypoxia-induced EPO expression in FAIK cells, confirming the repressive effect of methylation induced by SV40 large T antigen [3, 19]. A second cell line, REP cell-derived immortalized and cultivable cell line (Replic cells), is obtained by isolation of primary REP cells from a gene-modified mouse line (ISAM-REC) and then immortalized by lentiviral transduction of human *HRAS* gene [20]. Interestingly, Replic cells exhibit myofibroblastic phenotypes due to autonomous TGF-β1 signaling and lose the EPO production [1–3, 20]. Hypermethylation of *Epo* gene in Replic cells is also demonstrated, a finding in line with the possible consequence of overexpressing human RAS [19, 20, 34, 35].

Renznikoff et al. have reported the establishment of C3H10T1/2 (hereafter referred to as 10T1/2) cell line from C3H mouse embryo [36]. Previous study has shown that 10T1/2 cells can be differentiated to pericytes to support the formation of capillary-like structure in three-dimensional co-culture with endothelial cells [37]. The findings that 10T1/2 cells can stabilize microvasculature and transit to myofibroblasts are similar to that we demonstrate in the primary culture of kidney pericytes [3, 8, 9, 38, 39]. We therefore studied the properties of EPO expression in 10T1/2 cells and used murine model to prove the findings from cell line study.

## Materials and methods

### Cell culture

Mouse fibroblast cell lines, 10T1/2 (ATCC CCL-226) and NIH/3T3 (hereafter referred to as 3T3, ATCC CRL-1658) were maintained in DMEM/F12 (Invitrogen, Carlsbad, CA) supplemented with 10% fetal bovine serum (FBS, Hyclone, Marlborough, MA). In hypoxia experiments, cells were placed in an incubator with 21% O_2_ or in a hypoxia chamber (INVIVO2 200, Ruskinn Technology Ltd., Bridgend, UK) with 0.5% O_2_ for indicated duration. Cellular RNA was harvested by adding the TRIzol reagent (Invitrogen) or RNeasy mini kit (Qiagen, Valencia, CA) immediately after cells were taken out of the incubator and the supernatant was removed for storage. In roxadustat (MedChemExpress LLC, Monmouth Junction, NJ) experiments, cells were washed with 1 × PBS and renewed culture medium with or without roxadustat (50 µM). Cellular RNA was harvested at indicated time points. In TGF-β1 stimulation experiments, cells were washed with 1 × PBS and renewed culture medium with or without 5 ng/mL TGF-β1 (R&D Systems, Minneapolis, MN) in the presence of 5 µg/mL TGF-β receptor-1 (TGF-βR1) activin receptor-like kinase-5 (ALK5) inhibitor (ALK5i) SB431542 (Tocris Bioscience, Bristol, UK) or vehicle. Cellular RNA were harvested at indicated time points.

### Quantitative polymerase chain reaction (PCR)

The purity of RNA sample was determined based on the ratio of A260 to A280. cDNA was synthesized using the iScript cDNA Synthesis Kit (Bio-Rad, Hercules, CA). Quantitative PCR was performed using methods described previously [40]. The specific primer pairs used for PCR were listed in Additional file 1: Table S1.

### Western blot analysis

Whole cell lysates were prepared using radioimmunoprecipitation assay (RIPA) buffer supplemented with Protease Inhibitor Cocktail (Roche, Mannheim, Germany) and Phosphatase Inhibitor Cocktail (Roche). For HIF protein, cells were harvested by RIPA buffer in hypoxia chamber. Forty micrograms of cell lysate were heated at 95 °C for 8 min, applied to sodium dodecyl sulphate–polyacrylamide (7.5%) gel electrophoresis. A prestained marker was also electrophoresed as a molecular weight marker. The proteins were then transferred onto a polyvinylidine fluoride membrane (Millipore, Burlington, MA) using a transblot chamber with Tris buffer. Western blots were incubated at 4 °C overnight with primary antibodies. The next morning, membranes were washed with TBS/Tween-20 at room temperature 3 times for 5 min each and incubated with horseradish peroxidase (HRP)-conjugated secondary antibodies at room temperature for two hours. After washing, the membranes were incubated with Immobilon Classico Western HRP substrate (Millipore) according to the manufacturer’s instructions. The following primary antibodies were used to detect protein: HIF1α (PAB12138; Abnova, Taipei, Taiwan), HIF2α (C150132; LSBio, Seattle, WA), p-Smad2/3 (SC-11769-R; Santa Cruz Biotechnology, Santa Cruz, CA), total Smad2/3 (3102; Cell Signaling Technology, Danvers, MA), α-tubulin (ab176560; Abcam, Cambridge, UK), β-actin (4967; Cell Signaling), GAPDH (Ab9485; Abcam) and proliferating cell nuclear antigen (PCNA, RB-9055; Thermo Scientific, Fremont, CA).

### Transfection

Small interfering RNA (siRNA) experiments were carried out with Dharmafect transfection reagent (Dharmacon, Lafayette, CO), according to the manufacturer’s protocol. siRNA oligos were obtained as SMART-pools from Dharmacon. Silencing of *Hif1a* and/or *Epas1* were performed by transfection of ON-TARGET plus Mouse *Hif1a* and *Epas1* siRNA (5 nM). As control, non-targeting siRNA was used.

### Detection of EPO in culture media

Cell culture supernatant were stored in a − 80 °C freezer after collection, transferred into a − 20 °C freezer 12–16 h prior to analysis and thawed on ice before analysis. The analysis was performed according to the protocol of provided in the Mouse Erythropoietin Quantikine ELISA Kit (R&D Systems).

### Chromatin immunoprecipitation (ChIP)

ChIP was performed using a ChIP kit (Magna ChIP A/G, Millipore) according to the manufacturer’s protocol. Protein-DNA complexes were cross-linked by incubating 10T1/2 cells with 1% formaldehyde for 10 min. Glycine was added for 5 min to quench formaldehyde. Nuclear extraction using cell lysis buffer and nuclear lysis buffer supplemented with Protease Inhibitor Cocktail and MG-132 (Millipore) were sonicated to shear the chromatin to an average length of about 100 ~ 500 bp. Then the lysates were centrifuged for 10 min and supernatant was collected. Anti-HIF1α (NB100-449), anti-HIF2α (NB100-122, Novus Biologicals, Centennial, CO) antibody, or Rabbit IgG (SC-3888, Santa Cruz Biotechnology) were added with magnetic beads in the supernatant and incubated overnight at 4 °C with rotation. The magnetic bead–antibody/chromatin complex was washed and separated by magnetic separator. DNA was purified and amplified by PCR with primers listed in Additional file 1: Table S2. The PCR products were analyzed by electrophoresis and shown as a virtual gel. Quantitative PCR was performed using methods described previously [40]. The result was shown after normalization by the expression using input DNA as the PCR template.

### Methylation specific PCR (MSP)

Genomic DNA was prepared using DNeasy Blood & Tissue Kits (Qiagen). Sodium bisulfite conversion of genomic DNA was performed using the EZ DNA Methylation Kit according to the manufacturer’s protocol (ZYMO Research, Irvine, CA). PCR using bisulfite-converted genomic DNA was amplified with methylation-specific or unmethylation-specific primer pairs listed in Additional file 1: Table S3. Unmethylated and methylated controls were from mouse sperm genomic DNA and Methylated Mouse Genomic DNA Standard (ZYMO Research), respectively. The PCR products were analyzed by electrophoresis. The electrophoresis result was shown as a virtual gel. The percentage of methylation of *Epo* 5′-flanking region or *Epas1* 5′-flanking region for the indicated cells was determined by densitometric analysis of MSP products (methylated products divided by the sum of methylated and unmethylated products).

### Animals

C57BL/6 wild type (WT) mice were obtained from The Jackson Laboratory (Bar Harbor, ME). *Col1a1-GFP*^*Tg*^ mice were generated in the C57BL/6 background as previously described [10]. Adult (8–10 weeks) mice were used for all experiments. Kidney samples for mRNA analyses were obtained. In mouse models of kidney fibrosis experiments, unilateral ureteral obstruction (UUO) was performed in adult mice as previously described [40]. Briefly, the left ureter was ligated twice using 4–0 nylon surgical sutures at the level of the lower pole of kidney. For experiments of blocking TGF-β1 signaling in vivo, mice were injected intraperitoneally with the ALK5i SB431542 (5 mg/kg per day) (Tocris Bioscience) 2 h before surgery and then as scheduled until euthanasia at day 4. Col1a1-GFP^+^ pericytes and myofibroblasts were purified from kidneys before and after UUO surgery respectively using a method described previously [3]. Briefly, the kidney was decapsulated, diced and then incubated at 37ºC for 40 min with collagenase (50 mg/µl, Roche) and dispase (0.3 U/µl, Roche) in Hank’s Balanced Salt Solution (Sigma-Aldrich, St. Louis, MO). After centrifugation, cells were resuspended in 3 mL of PBS/1% BSA and filtered (40 µm). Col1a1-GFP^+^ pericytes and myofibroblasts were isolated from the single-cell preparation by sorting GFP^+^F4/80-APC^–^CD31-APC^–^CD324-APC^–^ cells using a FACSAria cell sorter (BD Biosciences, San Jose, CA).

### Statistics

Data were expressed as the mean ± standard error of the mean (SEM). One-way ANOVA with post hoc Tukey’s correction was used for the comparison between each group. Two-tailed Student’s t-test was used to compare two different groups. A P value < 0.05 was considered significant. Statistical analyses were carried out using the GraphPad Prism software (GraphPad Software, La Jolla, CA).

## Results

### 10T1/2 cells exhibited a fibroblastic phenotype

**Fig. 1:**
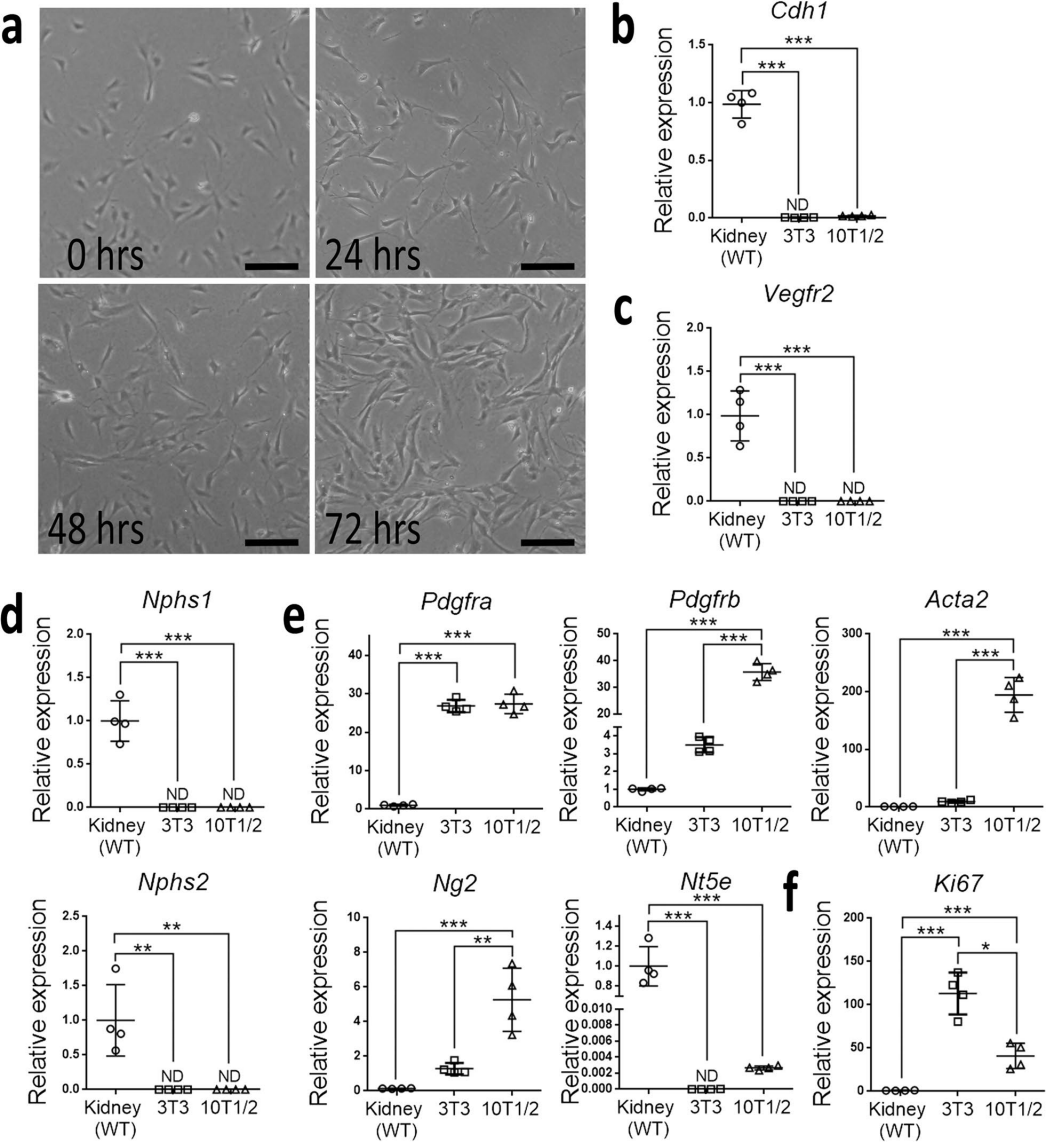
C3H10T1/2 cells exhibit a fibroblastic phenotype. **a** Bright-field images of C3H10T1/2 (referred to as 10T1/2) cells on cell culture dishes at the indicated time points after culture. Original magnification, ×400. Scale bar: 200 µm. **b**–**f** The relative mRNA expression normalized by *Hprt*. *Cdh1* encodes the epithelial cell marker E-cadherin; *Vegfr2* encodes the endothelial cell marker vascular endothelial growth factor (VEGF) receptor 2; *Nphs1* and *Nphs2* encode the podocyte markers nephrin and podocin, respectively; *Pdgfra*, *Pdgfrb, Acta2, Ng2* and *Nt5e* encode the fibroblast markers, platelet-derived growth factor receptor (PDGFR) α, PDGFRβ, α-smooth muscle actin (α-SMA), neuron-glial antigen 2 and ecto-5′-nucleotidase, respectively; *Ki67* encodes the marker for the assessment of cellular proliferative activity, Ki67 antigen; *Hprt* encodes hypoxanthine–guanine phosphoribosyl transferase and serves as the internal control. The gene expression of 10T1/2 cells is compared with those of 3T3 cells and the kidney from C57BL/6 wild type (WT) mice. The average expression of the kidney is set at 1. ND, not detected. The data are expressed as the mean ± standard error of the mean (SEM). n = 4 per group. *P < 0.05, **P < 0.01, ***P < 0.001 by one-way ANOVA with Tukey’s test

10T1/2 cells displayed fibroblastic morphology on cell culture dishes (Fig. 1a). To study the cell type-specific expression, we performed quantitative PCR and compared the gene expression to that of 3T3 fibroblasts (Fig. 1b–e). Kidney was used as a control and its gene expression reflected the composition of multiple cell types. Similar to 3T3 fibroblasts, 10T1/2 cells did not express *Cdh1*, *Vegfr2*, *Nphs1* and *Nphs2* which encoded E-cadherin, vascular endothelial growth factor receptor 2, nephrin and podocin, respectively (Fig. 1b–d). Both 3T3 and 10T1/2 cells expressed *Pdgfra* and *Pdgfrb* which encoded PDGFRα and PDGFRβ, respectively (Fig. 1e). Compared to 3T3 cells, 10T1/2 cells notably expressed higher levels of *Acta2*, *Ng2* and *Nt5e* which encoded α-SMA, neuron-glial antigen 2 and ecto-5’-nucleotidase, respectively (Fig. 1e). We also studied the marker for cell proliferation and demonstrated that 10T1/2 cells expressed lower level of *Ki67* (Fig. 1f). Nevertheless, both 3T3 and 10T1/2 cells could grow to confluence on cell culture dishes.

### 10T1/2 cells produced EPO in hypoxia and under PHD inhibition

**Fig. 2:**
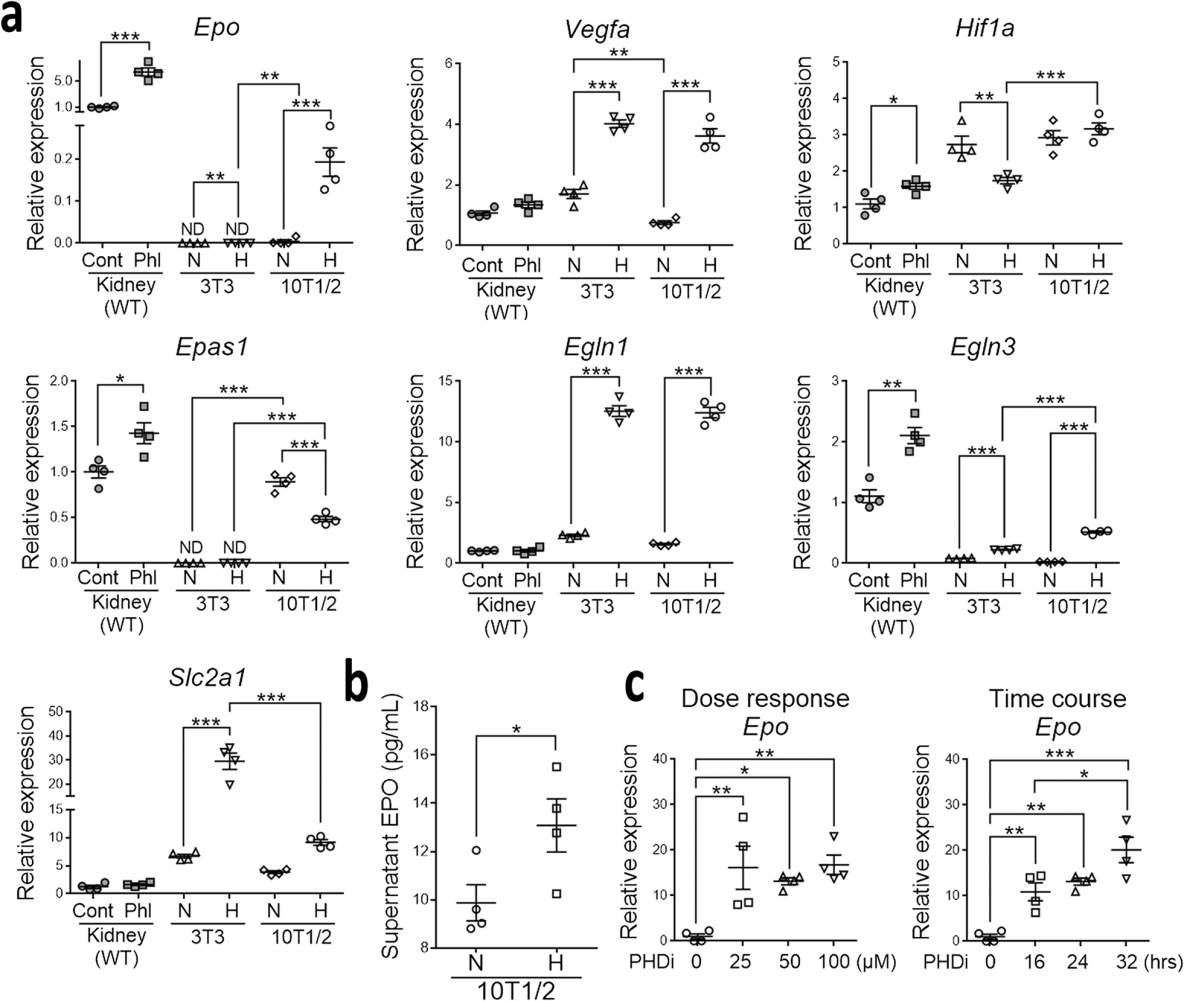
10T1/2 cells produce erythropoietin. **a** The relative mRNA expression normalized by *Hprt*. *Epo*, *Vegfa*, *Hif1a*, *Epas1*, *Egln1*, *Egln3* and *Slc2a1* encode erythropoietin (EPO), VEGF-A, hypoxia-inducible factor 1α (HIF1α), HIF2α, prolyl hydroxylase domain (PHD) 2, PHD3 and solute carrier family 2 member 1, respectively. 10T1/2 cells and 3T3 cells are analyzed after 24-h exposure to normoxia (N, 21% O_2_) or hypoxia (H, 0.5% O_2_). The mRNA expression of each gene in cells are compared to that of the kidney from the control mice (Cont) and those one day after phlebotomy (Phl). The average gene expression level of kidney from Cont mice is set at 1. n = 4 per group. ND, not detected. **b** EPO concentration in the supernatant of 10T1/2 cells after 24-h exposure to normoxia (N) or hypoxia (H). n = 4 per group. **c** The relative *Epo* expression normalized by *Hprt* in 10T1/2 cells after 24-h exposure to different concentrations of PHD inhibitor (PHDi, roxadustat) (left panel) or after exposure to PHDi (roxadustat 50 µM) for the indicated durations (right panel). n = 4 per group. Data are expressed as the mean ± SEM. *P < 0.05, **P < 0.01, ***P < 0.001 by one-way ANOVA with Tukey’s test for cell experiments in **a** and **c**, Student’s t test for animal experiments in **a** and cell experiments in **b**

Multiple genes are regulated by hypoxia in order to induce adaptive response. In addition to *Epo*, *Hif1a* and *Epas1*, we studied the common hypoxia response genes including *Vegfa*, *Egln1*, *Egln3* and *Slc2a1*. Compared to those in the chambers with 21% O_2_, 10T1/2 cells in the chambers with 0.5% O_2_ substantially increased the expression of *Epo*, *Vegfa*, *Egln1* and *Egln3* which encoded EPO, vascular endothelial growth factor-A (VEGF-A), prolyl hydroxylase domain (PHD) 2 and PHD3, respectively (Fig. 2a). The expression of *Hif1a* which encoded HIF1α was not changed by hypoxia, but in contrast, the expression of *Epas1* which encoded HIF2α was drastically decreased (Fig. 2a). Interestingly, *Epo* and *Epas1* were not detected in 3T3 cells (Fig. 2a). In contrast to the upregulation of *Slc2a1* which encoded solute carrier family 2 member 1 in 3T3 cells by hypoxia, the expression of *Slc2a1* in 10T1/2 cells was not changed by hypoxia (Fig. 2a). It was worth noting that *Epo* expression in 10T1/2 cells was extremely low when compared to that of REP pericytes in normal kidneys (Fig. 2a). Increased EPO was demonstrated in the supernatant of 10T1/2 cells in hypoxia (Fig. 2b). In addition to hypoxia, the PHD inhibitor (PHDi) roxadustat also increased *Epo* expression in 10T1/2 cells substantially (Fig. 2c).

### Hypoxia induced *Epo* through HIF2α in 10T1/2 cells

**Fig. 3:**
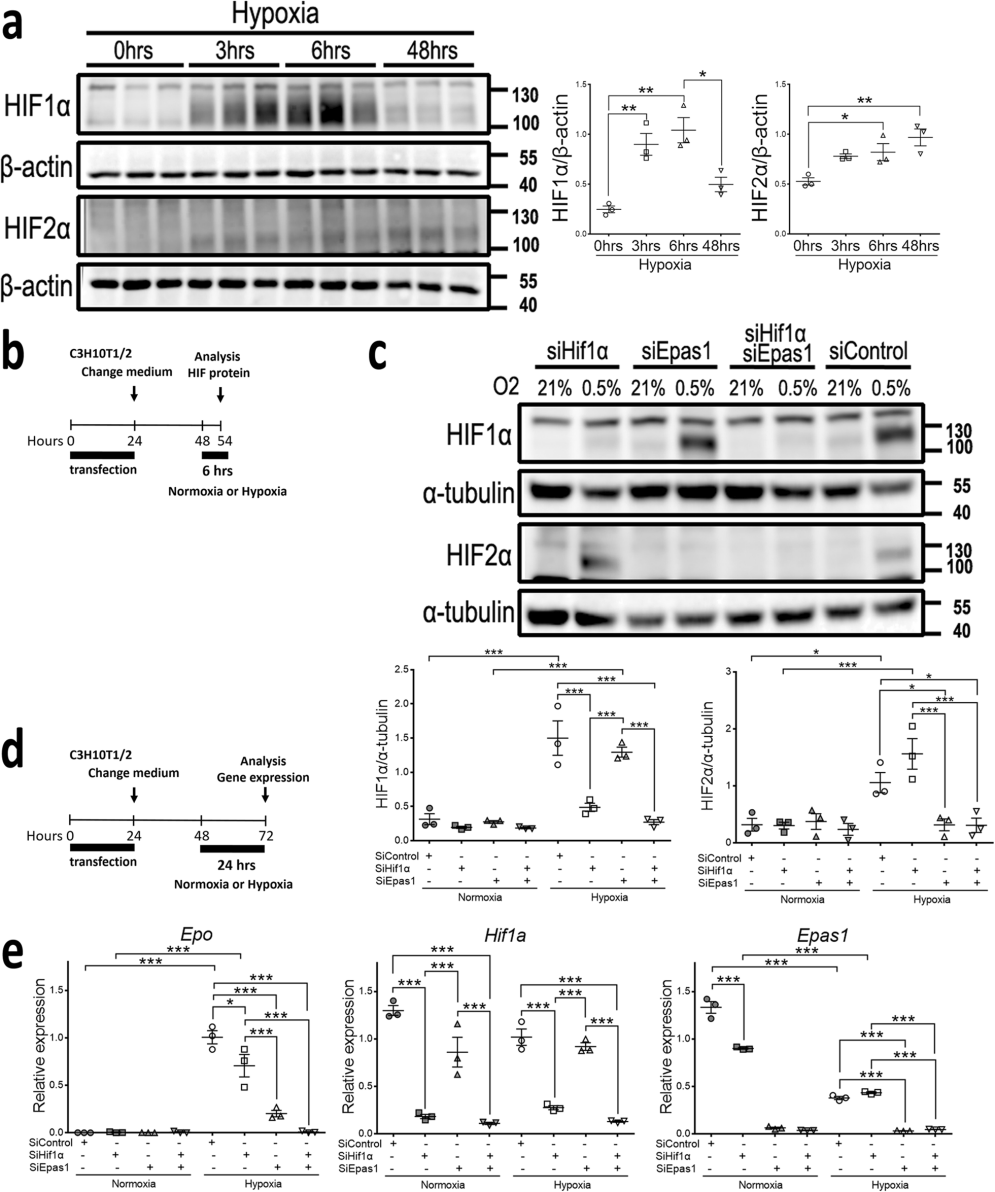
Hypoxia induces *Epo* through HIF2α in 10T1/2 cells. **a** Representative Western blot analysis for the expression of HIF1α and HIF2α in hypoxia chamber (0.5% O_2_) for the indicated duration. Right panels showing the expression of HIF1α and HIF2α normalized by β*-*actin. n = 3 per group. **b** Schema illustrating the siRNA transfection specific for *Hif1a* (siHif1a), *Epas1* (siEpas1) or control (siControl) and culture medium refresh. HIFs are analyzed after 6-h exposure to normoxia or hypoxia. **c** Upper panel showing the representative Western blot analysis for the expression of HIF1α and HIF2α after siRNA transfection and exposure to normoxia/hypoxia as indicated. Lower panels showing the expression of HIF1α and HIF2α normalized by α*-*tubulin. n = 3 per group. **d** Schema illustrating the siRNA transfection and culture medium refresh. Gene expression is analyzed after 24-h exposure to normoxia or hypoxia. **d** The relative mRNA expression of *Epo, Hif1a* and *Epas1* normalized by *Hprt*. n = 3 per group. Data are expressed as the mean ± SEM. *P < 0.05, **P < 0.01, ***P < 0.001 by one-way ANOVA with Tukey’s test in **a**, **c** and **e**

**Fig. 4:**
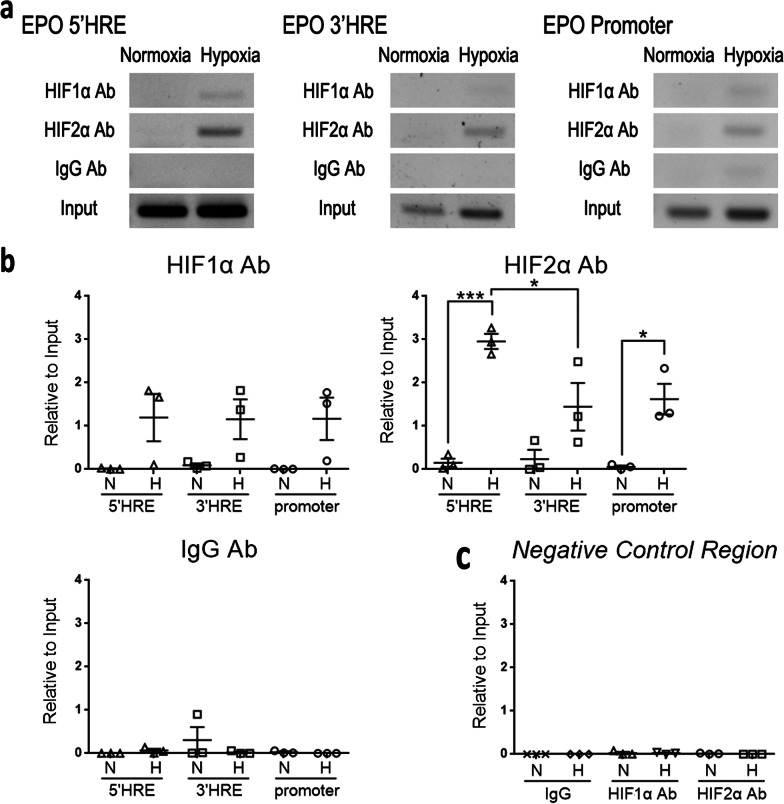
Hypoxia induces binding of HIF2α to distal 5’-enhancer of the *Epo* gene in 10T1/2 cells. **a** Representative electrophoresis of chromatin immunoprecipitation (ChIP)-polymerase chain reaction (PCR). 10T1/2 cells after 24-h exposure to normoxia or hypoxia are used for ChIP by specific antibody for HIF1α, HIF2α, or control IgG antibody (Ab). Precipitated chromatin is then studied by PCR using primers for hypoxia responsive element (HRE)^+^ 5′-enhancer (5′HRE), HRE^+^ 3′-enhancer (3′HRE) or promoter regions. **b** Dot charts showing the quantification of ChIP-quantitative PCR using SYBR green method. Precipitated chromatin is from **a** and the result is shown after normalization by the expression using input DNA as the PCR template. **c** Dot chart showing the quantification of ChIP-quantitative PCR using primers for the negative control region at 6.5 kb upstream of transcription starting site. Precipitated chromatin is from **a** and the result is also shown after normalization by the expression using input DNA as the PCR template. N, Normoxia; H, Hypoxia. The data are expressed as the mean ± SEM. n = 3 per group. *P < 0.05, **P < 0.01, ***P < 0.001 by one-way ANOVA with Tukey’s test

Both HIF1α and HIF2α increased expression in 10T1/2 cells after 3-h exposure to hypoxia (Fig. 3a) or PHDi (Additional file 2: Fig. S1). We transfected siRNA for *Hif1a* (siHif1a) or *Epas1* (siEpas1) to study the role of HIF1α and HIF2α in *Epo* induction by hypoxia. SiHif1a and siEpas1 specifically decreased the expression of *Hif1a*/HIF1α and *Epas1*/HIF2α in hypoxia, respectively (Fig. 3b–e). SiHif1a decreased the induction of *Epo*, *Egln1*, *Vegfa* and *Slc2a1* in hypoxia, whereas siEpas1 decreased the induction of *Epo, Egln3* and *Vegfa* in hypoxia (Fig. 3e, Additional file 2: Fig. S2). Notably, the inhibitory effect of HIF2α knockdown on *Epo* induction was substantially higher and not augmented by concomitant HIF1α knockdown (Fig. 3e), but both HIF1α knockdown and HIF2α knockdown contributed to the inhibitory effect on *Vegfa* induction (Additional file 2: Fig. S2). Using antibodies specific for HIF1α or HIF2α to precipitate chromatin, we demonstrated that hypoxia induced the most apparent binding of HIF2α to distal 5′-enhancer of *Epo* gene (Fig. 4a). Quantitative PCR of the precipitated chromatin confirmed that hypoxia induced significant binding of HIF2α to distal 5′-enhancer and promoter, but not 3′-enhancer (Fig. 4b). In contrast, hypoxia did not induce significant binding of HIF1α to distal 5′-enhancer, promoter and 3′-enhancer (Fig. 4b). Region at 6.5 kb upstream of the *Epo* transcription starting site served as the negative control (Fig. 4c). These data demonstrated HIF2α as the major transcriptional factor for hypoxia-induced *Epo* expression in 10T1/2 cells.

### Low methylation in 5′-flanking regions of ***Epo ***and ***Epas1*** genes in 10T1/2 cells

**Fig. 5:**
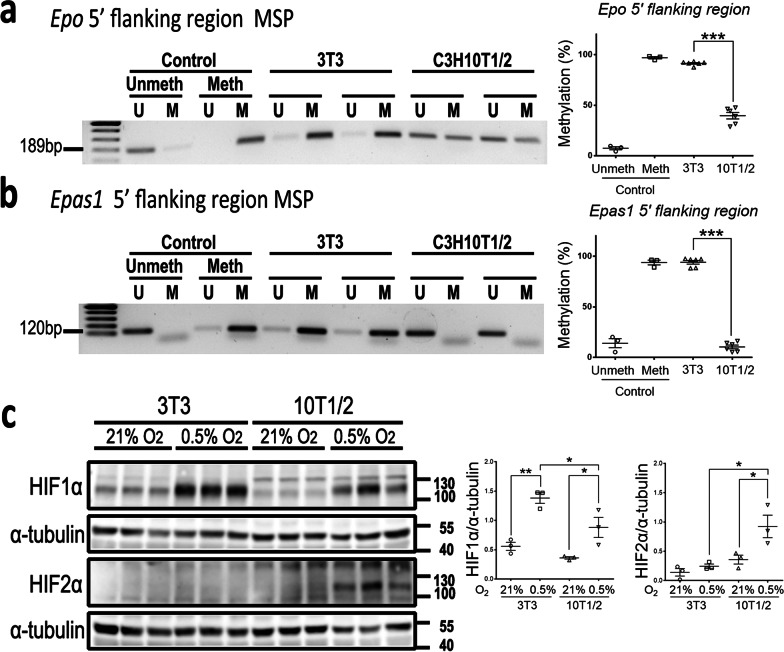
Low methylation in 5’-flanking regions of *Epo* and *Epas1* genes in 10T1/2 cells. **a**, **b** Representative electrophoresis of methylation specific PCR (MSP) using primers for the unmethylated (U) and methylated (M) 5′-flanking regions of *Epo* gene and *Epas1* gene from 3 independent experiments. Right panels showing the percentage of 5′-flanking region methylation determined by the densitometric analysis of the MSP products. The methylation levels in 3T3 cells are used for comparison. Data are expressed as the mean ± SEM. **P < 0.01, ***P < 0.001 by Student’s t test. Meth, methylated; Unmeth, unmethylated controls. **c** Representative Western blot analysis for HIF1α, HIF2α and α-tubulin in 10T1/2 and 3T3 cells after 6-h exposure to normoxia (21% O_2_) or hypoxia (0.5% O_2_). Right panels showing the expression of HIF1α and HIF2α which is normalized by α*-*tubulin. n = 3 per group. Data are expressed as the mean ± SEM. *P < 0.05, **P < 0.01 by one-way ANOVA with Tukey’s test

Since 10T1/2 cells, not 3T3 cells, expressed *Epo* and *Epas1*, we were curious about their epigenetic difference in *Epo* and *Epas1* genes. We focused on the methylation of *Epo* and *Epas1* gene in this study. Using MSP, we demonstrated hypermethylation in 5′-flanking regions of *Epo* and *Epas1* genes in 3T3 cells at the level comparable to the methylated control (Fig. 5a, b). The levels of methylation in *Epo* and *Epas1* genes were substantially lower in 10T1/2 cells (Fig. 5a, b). In addition to undetectable *Epas1* mRNA, we further confirmed that HIF2α protein could not be induced by hypoxia in 3T3 cells (Fig. 5c).

### 10T1/2 cells exhibited higher level of TGF-β1-ALK5 signaling and pro-fibrotic phenotype

Since REP pericytes possess cellular plasticity for myofibroblast transition when the kidney is damaged and TGF-β1 signaling is activated [2, 3], we studied the TGF-β1 signaling in both 10T1/2 and 3T3 cells. Compared to 3T3 cells, 10T1/2 cells expressed higher levels of *Tgfbr1* and *Tgfbr2* which encoded TGF-βR1 and TGF-βR2, respectively (Additional file 2: Fig. S3a). Western blot analysis revealed higher levels of phosphorylated SMAD2/3 in 10T1/2 (Additional file 2: Fig. S3b), a possible mechanism responsible for the higher expression of *Acta2*, *Fn1*, *Col1a1* and *Col3a1* which encoded α-SMA, fibronectin, type 1 collagen (α1 chain) and type 3 collagen (α1 chain), respectively (Fig. 1e, Additional file 2: Fig. S3c). However, the expression of *Serpine1*, which encoded plasminogen activator inhibitor-1, was lower in 10T1/2 cells (Additional file 2: Fig. S3c). The expression levels of *Acta2*, *Fn1*, *Col1a1* and *Serpine1* were extremely high in both 3T3 cells and 10T1/2 cells (Additional file 2: Fig. S3c), possibly because 3T3 cells and 10T1/2 cells are fibroblasts and kidneys are composed of heterogenous cell types.

**Fig. 6:**
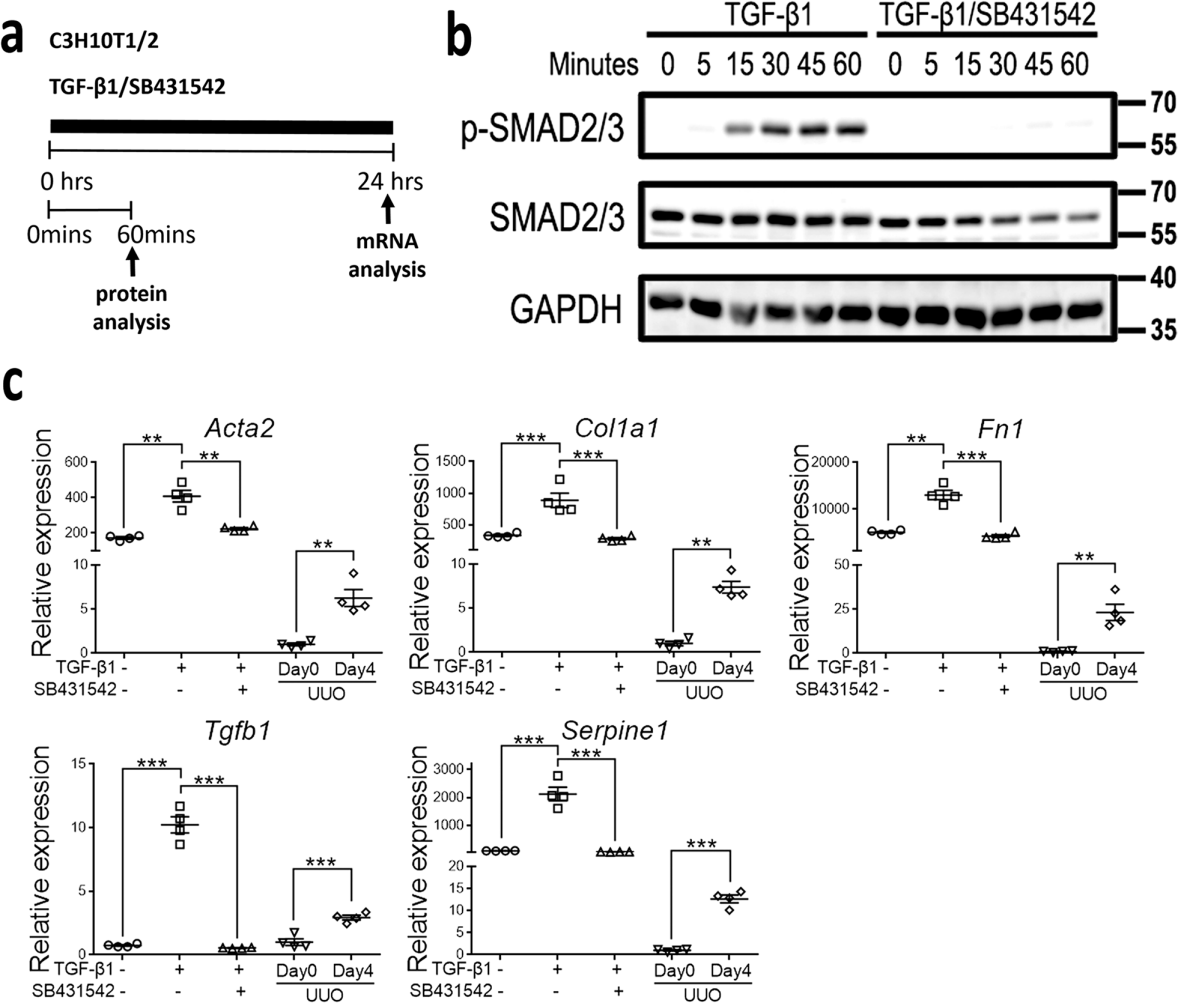
TGF-β1 stimulates pro-fibrotic phenotype of 10T1/2 cells through activin receptor-like kinase-5. **a** Schema illustrating the culture of 10T1/2 cells in medium with or without TGF-β1 (5 ng/mL) and activin receptor-like kinase-5 (ALK5) inhibitor (ALK5i) SB431542 (5 µg/mL). **b** Representative western blot analysis for p-SMAD2/3, SMAD2/3 and GAPDH at time points as indicated after treatments. **c** The relative mRNA expression normalized by *Hprt* at 24 h after treatments. The mRNA expression of each gene in cells are compared to that of the kidney from day 0 (normal kidney) and day 4 after unilateral ureteral obstruction (UUO kidney) surgery. Data are expressed as the mean ± SEM. The mean expression of normal kidney is set at 1. n = 4 per group. **P < 0.01, ***P < 0.001 by one-way ANOVA with Tukey’s test between cells and Student’s t test between normal and UUO kidneys

Activation of downstream signaling in 10T1/2 cells was demonstrated by the prompt phosphorylation of Smad2/3 after exposure to TGF-β1, which could be inhibited by ALK5i SB431542 (Fig. 6a, b). TGF-β1-stimulated 10T1/2 cells became more pro-fibrotic through upregulation of *Acta2*, *Col1a1*, *Fn1*, *Tgfb1* and *Serpine1*, which was also reversed by ALK5i SB431542 (Fig. 6a, c). UUO injury increased expression of pro-fibrotic genes in the kidneys (Fig. 6c). Although the expression levels of these pro-fibrotic genes in kidneys were extremely lower than those in 10T1/2 cells, the heterogenous cell components in kidneys made the comparison between kidneys and 10T1/2 cells unsuitable.

### TGF-β1 repressed *Epas1*-*Epo* axis through ALK5 in 10T1/2 cells

**Fig. 7:**
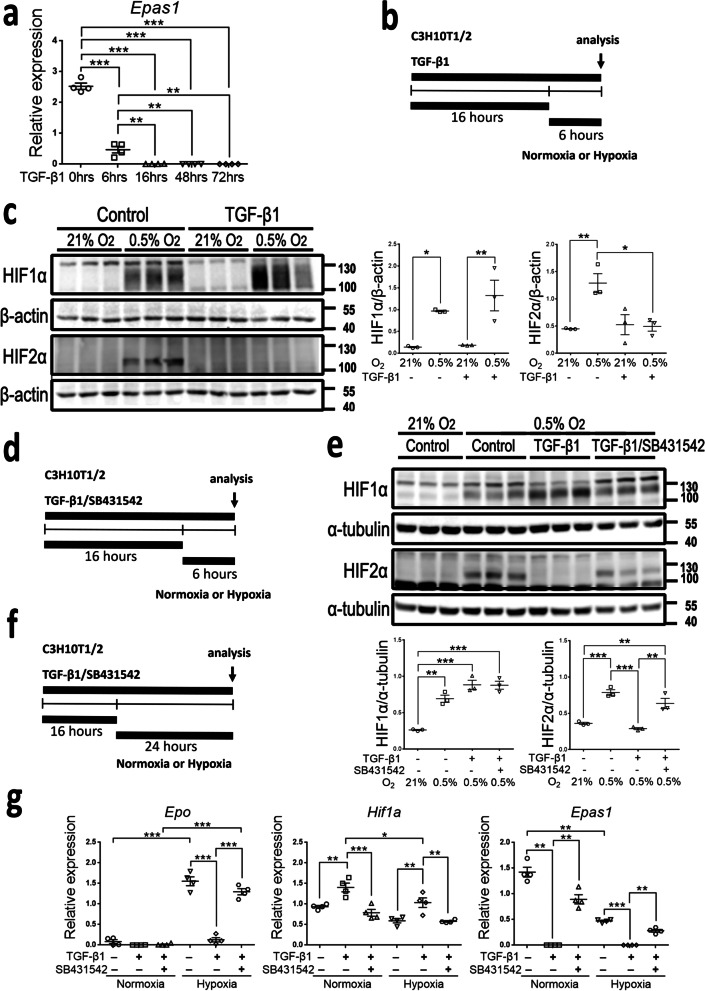
TGF-β1 inhibits HIF2α and *Epo* expression through ALK5 in 10T1/2 cells. **a** The relative *Epas1* expression normalized by *Hprt* in cells after exposure to TGF-β1 for the indicated duration. **b** Schema illustrating the analysis of HIFs in cells after 6-h exposure to normoxia or hypoxia in the presence or absence of TGF-β1. **c** Representative Western blot analysis for HIF1α, HIF2α and β-actin in cells of the experiment in **b**. Lower panels showing the expression of HIF1α and HIF2α which is normalized by β-actin. n = 3 per group. **d** Schema illustrating the analysis of HIFs in cells after 6-h exposure to normoxia or hypoxia in the presence or absence of TGF-β1 with or without ALK5i SB431542. **e** Representative Western blot analysis for HIF1α, HIF2α and α*-*tubulin in cells of the experiment in **d**. Lower panels showing the expression of HIF1α and HIF2α which is normalized by α*-*tubulin. n = 3 per group. **f** Schema illustrating the analysis of gene expression in cells after 24-h exposure to normoxia or hypoxia in the presence or absence of TGF-β1 with or without ALK5i SB431542. **g** The relative mRNA expression of *Epo*, *Hif1a* and *Epas1* normalized by *Hprt* in cells of the experiment in **f**. n = 4 per group. Data are expressed as the mean ± SEM. *P < 0.05, **P < 0.01, ***P < 0.001 by one-way ANOVA with Tukey’s test

In addition to induce the pro-fibrotic phenotype of 10T1/2 cells (Fig. 6c), TGF-β1 inhibited *Epas1* expression substantially, an effect noted as early as after 6-h exposure to TGF-β1 (Fig. 7a). Therefore, we studied the effect of TGF-β1 on HIF expression in 10T1/2 cells (Fig. 7b, c, Additional file 2: Fig. S4a, b). TGF-β1 inhibited the expression of HIF2α, but not HIF1α induced by hypoxia or PHDi (Fig. 7c, Additional file 2: Fig. S4b). In the presence of ALK5i SB431542, the inhibitory effect of TGF-β1 on HIF2α expression induced by hypoxia or PHDi was abolished (Fig. 7d, e, Additional file 2: Fig. S4c, d). In addition, the inhibitory effect of TGF-β1 on *Epas1* expression was also ALK5 dependent (Fig. 7f, g). Meanwhile, hypoxia-induced nuclear accumulation of HIF2α was inhibited by TGF-β1 through ALK5 (Additional file 2: Fig. S5). In consequence, the induction of *Epo* in hypoxia was inhibited by TGF-β1 through ALK5 (Fig. 7f, g). In contrast, TGF-β1 increased the expression of *Hif1a* in normoxia and hypoxia through ALK5, but did not affect the expression of HIF1α (Fig. 7c, e, g). Moreover, TGF-β1 further increased the expression of *Egln1*, *Slc2a1* and *Vegfa* in hypoxia through ALK5 (Additional file 2: Fig. S6).

In contrast to our previous report on primary culture of kidney pericytes [3], TGF-β1 did not upregulate *Dnmt* genes which encoded DNMT in 10T1/2 cells even after 72-h exposure (Additional file 2: Fig. S7a, b). TGF-β1 did not change the methylation in 5′-flanking regions of *Epo* and *Epas1* genes (Additional file 2: Fig. S7c).

### Pro-fibrotic kidney injury downregulated the expression of *Epas1* and *Epo* in pericytes

**Fig. 8:**
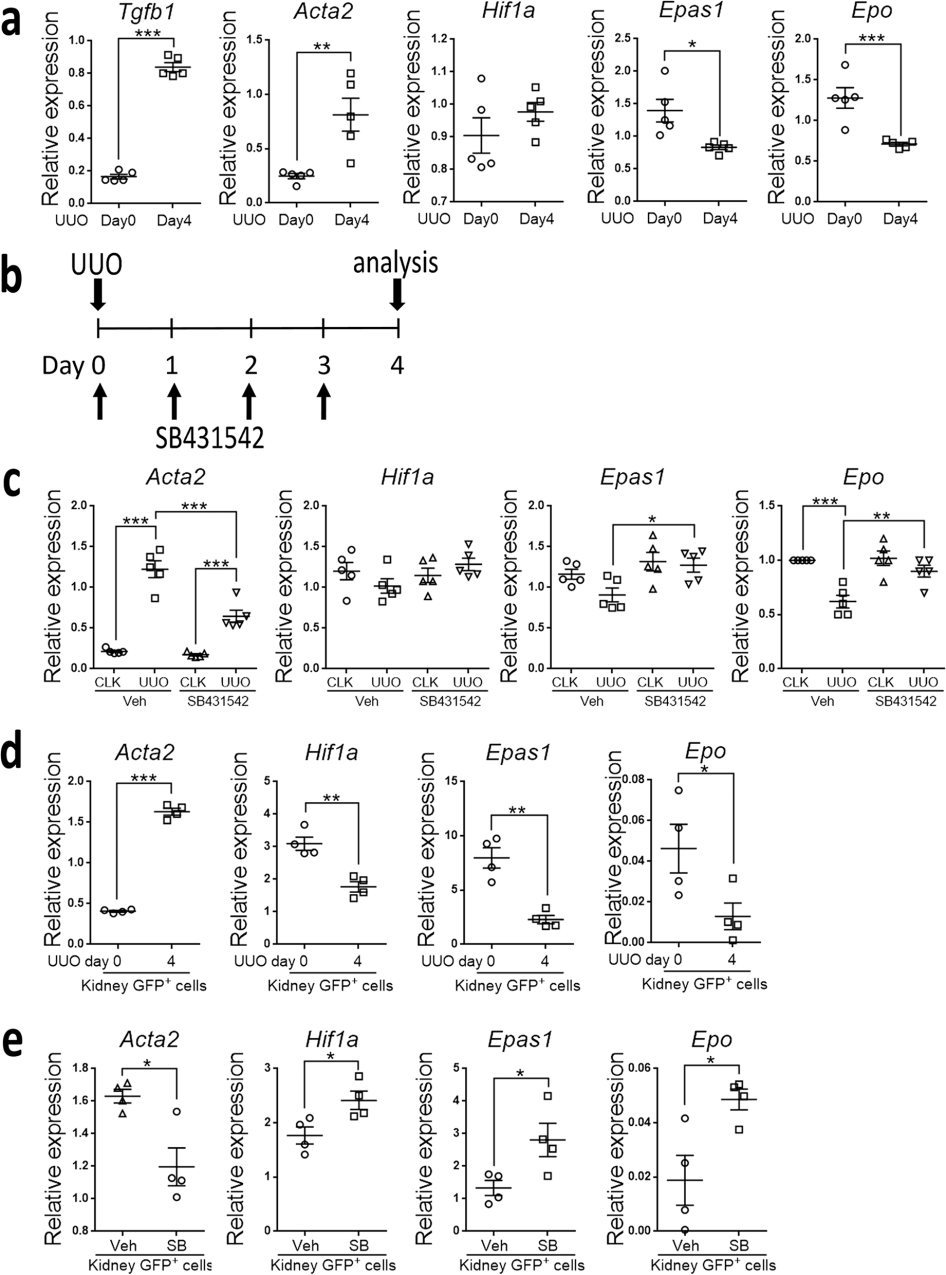
Pro-fibrotic injury downregulates expression of *Epas1* and *Epo* of pericytes through ALK5. **a** The relative mRNA expression of *Tgfb1, Acta2, Hif1a*, *Epas1* and *Epo* in the kidneys at day 0 (normal kidney) and day 4 after UUO surgery (UUO kidney). n = 5 at each time point. **b** Schema illustrating the analysis of kidneys and pericytes in mice with daily vehicle (Veh) or ALK5i SB431542 (5 mg/kg/day) treatment after UUO surgery. **c** The relative mRNA expression of *Acta2, Hif1a*, *Epas1* and *Epo* in the contralateral (CLK) and UUO kidneys of mice with daily Veh or ALK5i SB431542 treatment after UUO surgery according to the schema in **b**. n = 5 at each time point. **d** The relative mRNA expression of *Acta2, Hif1a*, *Epas1* and *Epo* in Col1a1-GFP^+^ pericytes isolated from kidneys before (day 0, normal kidney pericytes) and after (day 4 UUO kidney myofibroblasts) UUO surgery. n = 4 at each time point. **e** The relative mRNA expression of *Acta2, Hif1a*, *Epas1* and *Epo* of Col1a1-GFP^+^ myofibroblasts isolated from kidneys of mice with daily Veh or ALK5i SB431542 treatment at day 4 after UUO surgery according to the schema in **b**. n = 4 per group. Data are expressed as the mean ± SEM. *P < 0.05, **P < 0.01, ***P < 0.001 by Student’s t test in **a**, **d** and **e**, and by one-way ANOVA with Tukey’s test in **c**

Our previous study has shown a prompt activation of TGF-β1-Smad2/3 signaling in kidney pericytes after pro-fibrotic injury induced by UUO surgery [40]. Increased expression of *Tgfb1* and *Acta2* in the kidneys of WT mice after UUO surgery was demonstrated again (Fig. 8a). In contrast, the expression of *Epas1* and *Epo* was decreased in the kidneys after UUO surgery (Fig. 8a). In WT mice with ALK5i SB431542 treatment, the upregulation of *Acta2* while the downregulation of *Epas1* and *Epo* in the kidneys after UUO surgery was reversed (Fig. 8b, c). We then used *Col1a1-GFP*^*Tg*^ mice to isolate Col1a1-GFP^+^ pericytes and myofibroblasts from the kidneys before and after UUO surgery, respectively. Although the expression of *Acta2* was increased in myofibroblasts, the expression of *Hif1a*, *Epas1* and *Epo* was decreased in myofibroblasts (Fig. 8b, d). In *Col1a1-GFP*^*Tg*^ mice with ALK5i SB431542 treatment, the expression of *Acta2* in myofibroblasts was decreased while the expression of *Hif1a*, *Epas1* and *Epo* was increased (Fig. 8b, e), a mechanism supporting the inhibitory effect of TGF-β1-ALK5 signaling on *Epas1*-*Epo* axis in REP pericytes.

## Discussion

The main findings of this study included: (1) 10T1/2 cells represented a reliable cell line in the studies for EPO regulation and myofibroblast transition; (2) 10T1/2 cells expressed *Epo* through the binding of HIF2α to 5′-enhancer and promoter of *Epo* gene; (3) TGF-β1 induced myofibroblast transition and repressed *Epas1*-*Epo* through ALK5 in 10T1/2 cells; and (4) pro-fibrotic injury activated TGF-β1-ALK5 signaling and repressed *Epas1*-*Epo* in REP pericytes in vivo, confirming the findings in 10T1/2 cells in vitro.

10T1/2 cells did not express markers for epithelial cells, endothelial cells and podocytes. Similar to REP pericytes [3, 7, 9, 10, 12, 40], 10T1/2 cells expressed makers for mesenchymal cells. Compared to 3T3 cells, 10T1/2 cells expressed higher levels of *Pdgfrb*, *Ng2*, *Acta2*, *Col1a1*, *Col3a1* and *Fn1* which were expressed in REP pericytes [3, 7, 9, 10, 12, 40], suggesting 10T1/2 cells as a REP pericyte-like cell line. Previous studies have demonstrated that different subpopulations of kidney interstitial cells produce EPO and mostly are PDGFRβ^+^ [3, 4, 6, 19]. Interestingly, one of the reported REP cell lines, FAIK cells, exhibit telocyte-like phenotype [19]. 10T1/2 cells exhibited typical characteristics of fibroblasts. They did share features with telocytes including several prolongations and spindle shape of cell body. However, the main difference between 10T1/2 cells and telocytes was the cell processes. The processes of 10T1/2 cells were usually cone shape compared to those of telocytes which are moniliform aspect [41]. Besides, several studies indicate that renal telocytes are positive for CD34, CD117 and vimentin [42, 43]. We did not perform extensive transcriptomic analysis in 10T1/2 cells. However, it will be interesting to study whether 10T1/2 cells share telocyte-like gene expression profile in the future.

In addition to produce EPO and myofibroblast transition that we demonstrated in this study, evidence has shown that 10T/12 cells can stabilize microvasculature, an important property of pericytes [3, 8, 9, 37–39]. Compared to 3T3 cells which did not express *Epas1* and *Epo*, 10T1/2 cells showed substantially low methylation in 5’-flanking regions of *Epas1* and *Epo* genes, a finding supporting their capability as an EPO-producing cell line. Interestingly, HIF2α expression was increased but *Epas1* expression was drastically decreased by hypoxia in 10T1/2 cells. Initially, we proposed a negative feedback leading to downregulation of *Epas1* when HIF2α expression was increased by hypoxia. But we also found that hypoxia did not affect *Hif1a* expression even if HIF1α was upregulated. A human cancer cell study has shown that *HIF1A* and *EPAS1* transcriptional response to hypoxia varies among human cells [44]. So far, we could not conclude the mechanism responsible for *Epas1* downregulation by hypoxia in 10T1/2 cells. Besides, we demonstrated that hypoxia could upregulate *Slc2a1* expression in 3T3 cells, not in 10T1/2 cells. *Slc2a1* encodes solute carrier family 2 member 1 to enhance glucose transport in response to hypoxia [45]. The mechanism that 10T1/2 cells did not increase *Slc2a1* substantially in hypoxia needs further study.

Similar to REP pericytes, 10T1/2 cells expressed *Epo* through HIF2α-activated transcription [3, 28]. Although 10T1/2 cells were not derived from the REP cells directly, our data demonstrated the major binding site for HIF2α was the distal 5′-enhancer of *Epo* gene, a reported HRE located at KIE in the regulation of *Epo* expression [30]. FAIK cells and Replic cells are derived from the REP cells directly, but overexpression of SV40 large T antigen or human RAS results in hypermethylation and repression of *Epas1* and *Epo* genes [19, 20, 34, 35]. But similar to the findings in FAIK cells and Replic cells, 10T1/2 cells expressed extremely low level of *Epo* when compared to that of REP pericytes in normal kidneys. The mechanisms might be that 10T1/2 cells exhibited higher level of TGF-β1-ALK5 signaling and pro-fibrotic phenotype.

Sustained exposure to TGF-β1 has been shown to induce DNMT and hypermethylation of *Rasal1*, *Epo* and *Ybx2* in kidney pericytes [3, 9, 14]. In addition to the repressive effect of sustained TGF-β1 exposure through *Epo* hypermethylation [3], this study further demonstrated that short-term TGF-β1 exposure could downregulate *Epas1* and *Epo* of 10T1/2 cells in normoxia and hypoxia through an ALK5-dependent mechanism. In contrast to the inhibitory effect on the expression of *Epas1* and *Epo*, TGF-β1 increased the expression of *Hif1a*, *Egln1*, *Slc2a1* and *Vegfa* through ALK5 activation. *Egln1* upregulation might be involved in the downregulation of HIF2α by increasing protein degradation. Notably, Souma et al. demonstrated disrupted hypoxic response in the kidney after UUO surgery, possibly due to over-activation of PHD in REP cells [15, 20]. Our previous data that TGF-β1 signaling increases soon after UUO-induced fibrotic injury and TGF-β1 represses *Epo* through hypermethylation after 72-h exposure [3, 40], and our finding in this study that TGF-β1 could repress *Epas1*-*Epo* within 24-h exposure suggested that TGF-β1 could inhibit EPO expression in REP cells via a dual mechanism–first via direct transcriptional repression and then via methylation. The dual mechanism by which TGF-β1 inhibits gene expression seems common because TGF-β1 also inhibits *Rasal1* and *Ybx2* by the same mechanism [9, 14]. In the murine model of early renal fibrosis at day 4 after UUO surgery, we demonstrated that SB431542 could reverse *Epo* expression in the UUO kidney myofibroblasts through inhibiting TGF-β1-activated ALK5. Our finding could be supported by the recent report that selective disruption of TGF-βR2 in renal PDGFRβ^+^ cells preserves EPO production but no discernible effect on myofibroblast markers in the murine model of renal fibrosis [46].

In a personal communication with Norio Suzuki (Tohoku University), we knew that HIF2α was not detected in interstitial cells of UUO fibrotic kidneys despite worsening hypoxia. This study demonstrated that PHDi could not increase HIF2α expression of 10T1/2 cells in the presence of TGF-β1. However, PHDi emerges as a promising therapeutic agent for the renal anemia principally through stabilizing HIF [47–49]. One of the plausible reasons would be that a continuous spectrum existed between normal kidney pericytes and scar-producing myofibroblasts in fibrotic kidneys [3, 20]. In myofibroblasts with hypermethylation of *Epas1* and *Epo* genes, or with exposure to high TGF-β1, the effect of PHDi on HIF2α-EPO expression would be limited. This proposal could be supported by the elegant study of Bernhardt et al. that PHDi FG-2216 fails to increase EPO in 2 of 6 hemodialysis patients with native kidneys in situ [50]. Chen et al. has also shown that a hemoglobin response (i.e., an increase of ≥ 1.0 g/dL from baseline) occurs in 85 of 101 CKD patients in the PHDi roxadustat group by week 9 after starting clinical trial, implying a poor response in 16% of patients [47]. Based on evidence from previous and current studies [3, 19, 20, 47, 50], the demethylating agent and ALK5i might provide additive therapeutic effect on EPO production.

In previous reports using Hep3B cells as the model, Faquin et al. demonstrate an inhibitory effect of TGF-β1 on hypoxia-induced EPO production [51], but Sánchez-Elsner et al. show the opposite result [52]. Hep3B cells, different from REP cells, produce EPO through binding HIF to liver specific 3′-enhancer [26, 29]. Sánchez-Elsner et al. demonstrate the interaction between TGF-β1-activated Smad3/4 and HIF1α to enhance hypoxia-induced EPO production [52]. In contrast, we demonstrated the inhibitory effect of TGF-β1-ALK5 on *Epo* expression not only in 10T1/2 cells but also in murine fibrotic kidneys. To confirm the repressive effect of TGF-β1-ALK5 on kidney-specific *Epas1* or *Epo* regulation, we need to perform the reporter assay to study the repressive effect of Smad protein on the promoter/enhancer of *Epas1* and *Epo* genes in the future.

We did not find the hypermethylation of *Epas1* and *Epo* genes in 10T1/2 cells after 72-h exposure to TGF-β1, a finding different from our previous report on primary culture of kidney pericytes [3]. The lack of increased DNMT by TGF-β1 in 10T1/2 cells might be one of the plausible reasons and provide a chance to serve a REP cell-like cell line for EPO biology study.

## Conclusions

Based on our data, the clonal mouse embryo cell line 10T1/2 expresses EPO through the major binding of HIF2α to the 5′-HRE located at KIE of *Epo* gene. TGF-β1 not only promotes the transition of 10T1/2 cells to pro-fibrotic phenotype but also represses *Epas1*-*Epo* expression. 10T1/2 cells serve a REP pericyte-like model for EPO biology study.

## Supplementary Information

**Additional file 1.Table S1.** Primer sequences used in quantitative polymerase chain reaction. Table S2. Primer sequences used in (quantitative) chromatin immunoprecipitation polymerase chain reaction. Table S3. Primer sequences used in methylation-specific polymerase chain reaction of *Epo* and *Epas1* 5’ flanking regions.

**Additional file 2. Figure S1.** Prolyl hydroxylase domain inhibitor induces hypoxia-inducible factor 1α﻿ and hypoxia-inducible factor 2α in C3H10T1/2 cells. Figure S2. SiRNA transfection specific for *Hif1a* and *Epas1* downregulates hypoxia-induced genes. Figure S3. 10T1/2 cells exhibited higher levels of TGF-β1 signaling. Figure S4. TGF-β1 inhibits PHDi-induced HIF2α expression through activin receptor-like kinase-5 in 10T1/2 cells. Figure S5. TGF-﻿β﻿1 inhibits hypoxia-induced nuclear accumulation of HIF2α through ALK5 in 10T1/2 cells. Figure S6. TGF-﻿β﻿1 increases hypoxia-induced expression of *Egln1*, *Vegfa* and *Slc2a1* through ALK5 in 10T1/2 cells. Figure S7. TGF-﻿β﻿1 does not change methylation in 5’ flanking regions of *Epo* and *Epas1* genes in 10T1/2 cells.

## Data Availability

All materials are available by the corresponding author.

## Abbreviations

ALK5: Activin receptor-like kinase-5
ALK5i: ALK5 inhibitor
ANOVA: Analysis of variance
α-SMA: α-Smooth muscle actin
ChIP: Chromatin immunoprecipitation
DNMT: DNA methyltransferase
EPO: Erythropoietin
FAIK cell: Fibroblastoid atypical interstitial kidney cell
GFP: Green fluorescent protein
HIF: Hypoxia-inducible factor
HRE: Hypoxia response element
MSP: Methylation specific PCR
PDGFR: Platelet-derived growth factor receptor
PHD: Prolyl hydroxylase domain
PHDi: PHD inhibitor
REP cell: Renal EPO-producing cell
Replic cell: REP cell-derived immortalized and cultivable cell
SEM: Standard error of the mean
TGF-β1: Transforming growth factor-β1
UUO: Unilateral ureteral obstruction
VEGF-A: Vascular endothelial growth factor-A
3T3 cell: NIH/3T3 cell
10T1/2 cell: C3H10T1/2 cell
