# Supplementary material for: Transforming growth factor-β1 decreases erythropoietin production through repressing hypoxia-inducible factor 2α in erythropoietin-producing cells
Source: J Biomed Sci. 2021 Nov 2;28:73. doi: 10.1186/s12929-021-00770-2 (PMC8561873; doi:10.1186/s12929-021-00770-2)
Supplement: Supplementary file 1 — Additional file 1.Table S1. Primer sequences used in quantitative polymerase chain reaction. Table S2. Primer sequences used in (quantitative) chromatin immunoprecipitation polymerase chain reaction. Table S3. Primer sequences used in methylation-specific polymerase chain reaction of Epo and Epas1 5’ flanking regions. [file 12929_2021_770_MOESM1_ESM.pdf]

Shih et al. Addition file 1

Supplementary Table S1. Primer sequences used in quantitative polymerase chain reaction

Gene		Sequences
Cdh1	Forward	CAA GGA CAG CCT TCT TTT CG
	Reverse	TGG ACT TCA GCG TCA CTT TG
Vegfr2	Forward	TCT GGA CTC TCC CTG CCT AC
	Reverse	ATG CAA GGA CCA TCC CAC TG
Nphs1	Forward	CAT ATC GCC AAG CCT TCA CA
	Reverse	CAG GCC AGC GAA GGT CAT AG
Nphs2	Forward	TCT CCG TCT CCA GAC CTT GG
	Reverse	ATG GTG GTT TGC ACC AGG AA
Pdgfra	Forward	TGG CAT GAT GGT CGA TTC TA
	Reverse	CGC TGA GGT GGT AGA AGG AG
Pdgfrb	Forward	CAC CTT CTC CAG TGT GCT GA
	Reverse	GGA GTC CAT AGG GAG GAA GC
Acta2	Forward	CTG ACA GAG GCA CCA CTG AA
	Reverse	CAT CTC CAG AGT CCA GCA CA
Ng2	Forward	CTC GGT GCT GTC CAG ATT CA
	Reverse	AAC TGG AGC AGC AGG TCT AC
Nt5e	Forward	CGC TCA GAA AGT TCG AGG TGT G
	Reverse	CGC AGG CAC TTC TTT GGA AGG T
Ki67	Forward	CTG CCT GCG AAG AGA GCA TC
	Reverse	AGC TCC ACT TCG CCT TTT GG
Epo	Forward	GCC TCA CTT CAC TGC TTC GG
	Reverse	AGC AGG TGG GGT GGT ATC TG
Vegfa	Forward	ATC TTC AAG CCG TCC TGT GT
	Reverse	GCA TTC ACA TCT GCT GTG CT
Hif1a	Forward	ACA ACG CGG GCA CCG ATT CG
	Reverse	GCT CAC ATT GTG GGG AAG TGG C
Epas1	Forward	GGG CCA CGG CGA CAA TGA CA
	Reverse	GCT GAT GGC CAG GCG CAT GA
Egln1	Forward	GCG GGA AGC TGG GCA ACT ACA
	Reverse	CAT AGC CTG TTC CGT TGC CTG GG
Egln3	Forward	CTA TGT CAA GGA GCG GTC CAA
	Reverse	TAC AGC GGC CAT CAC CAT TG
Slc2a1	Forward	AGT GAC GAT CTG AGC TAC GG
	Reverse	CCA GTG TTA TAG CCG AAC TGC

Tgfb1	Forward	GGA CTC TCC ACC TGC AAG AC
	Reverse	GAC TGG CGA GCC TTA GTT TG
Acvrl1	Forward	GAC ATG ACT TCG CGG AAC TC
	Reverse	ACT CTT GAG GTC ACG ATG GG
Bmpr1b	Forward	GCG CTA TAT GCC TCC AGA AG
	Reverse	CTC CTT GCA ATC TCC CAG AG
Tgfbr1	Forward	TGC CAT AAC CGC ACT GTC A
	Reverse	AAT GAA AGG GCG ATC TAG TGA TG
Tgfbr2	Forward	ATC TGG AAA ACG TGG AGT CG
	Reverse	TCA CTT CTC CCA CAG CAT TG
Fn1	Forward	ATC CTG GCC TGG AGT ACA AC
	Reverse	AAG AGT TTA GCG GGG TCC AC
Col1a1	Forward	GAG CGG AGA GTA CTG GAT CG
	Reverse	GTT CGG GCT GAT GTA CCA GT
Col3a1	Forward	GCT TTG TGC AAA GTG GAA CCT
	Reverse	AGG TGG CTG CAT CCC AAT TC
Serpine1	Forward	AGT CTT TCC GAC CAA GAG CA
	Reverse	ATC ACT TGC CCC ATG AAG AG
Hprt	Forward	GGT TAA GCA GTA CAG CCC CA
	Reverse	TCC AAC ACT TCG AGA GGT CC
Dnmt1	Forward	CGG CTC AAA GAC TTG GAA AG
	Reverse	TAG CCA GGT AGC CTT CCT CA
Dnmt3a	Forward	ACC AGG CCA CCT ACA ACA AG
	Reverse	TGC TTG TTC TGC ACT TCC AC
Dnmt3b	Forward	ACT TGG TGA TTG GTG GAA GC
	Reverse	CCA GAA GAA TGG ACG GTT GT

Supplementary Table S2. Primer sequences used in (quantitative) chromatin immunoprecipitation polymerase chain reaction

Gene		Sequences
Epo	Forward	TTG TGT CTC CCT GCA CGT AT
5' HRE	Reverse	AGA AAC AAC AAA CGG AGG CC
Epo	Forward	TCC TAG CTG TAC CTC ACC CC
3' HRE	Reverse	TAT TGA CTA GCG TGG GCA GG
Epo	Forward	GCG CCC AAC TTT TCA TAG GTC
promoter	Reverse	CGC ATC TGA GAG ATT TGC GG
Negative control	Forward	ATC AAT CTG CCT GCC TCT CAC
region	Reverse	ACT GTA GCT GGG TGT AGG AG

Supplementary Table S3. Primer sequences used in methylation-specific polymerase chain reaction of *Epo* and *Epas1* 5' flanking regions

Gene			Sequences
Epo 5' flanking region	Unmethylation-specific	Forward	GTT GGT GGT TGT GTT TTA TTG TGT TTT T
		Reverse	AAA CTC CTT AAC AAC CCA AAA C
	Methylation-specific	Forward	CGG TGG TTG TGT TTT ATT GTG TTT TC
		Reverse	AAA CTC CTT AAC GAC CCG AAA
Epas1 5' flanking region	Unmethylation-specific	Forward	GAT AGT TGA GGA GGT TGG ATA TTT G
		Reverse	ACC ATA CAA TCT CAA AAC ACT ACC A
	Methylation-specific	Forward	ATA GTC GAG GAG GTC GGA TAT TC
		Reverse	CCA TAC AAT CTC AAA ACA CTA CCG
