# Supplementary material for: Transforming growth factor-β1 decreases erythropoietin production through repressing hypoxia-inducible factor 2α in erythropoietin-producing cells
Source: J Biomed Sci. 2021 Nov 2;28:73. doi: 10.1186/s12929-021-00770-2 (PMC8561873; doi:10.1186/s12929-021-00770-2)
Supplement: Supplementary file 2 — Additional file 2. Figure S1. Prolyl hydroxylase domain inhibitor induces hypoxia-inducible factor 1α﻿ and hypoxia-inducible factor 2α in C3H10T1/2 cells. Figure S2. SiRNA transfection specific for Hif1a and Epas1 downregulates hypoxia-induced genes. Figure S3. 10T1/2 cells exhibited higher levels of TGF-β1 signaling. Figure S4. TGF-β1 inhibits PHDi-induced HIF2α expression through activin receptor-like kinase-5 in 10T1/2 cells. Figure S5. TGF-﻿β﻿1 inhibits hypoxia-induced nuclear accumulation of HIF2α through ALK5 in 10T1/2 cells. Figure S6. TGF-﻿β﻿1 increases hypoxia-induced expression of Egln1, Vegfa and Slc2a1 through ALK5 in 10T1/2 cells. Figure S7. TGF-﻿β﻿1 does not change methylation in 5’ flanking regions of Epo and Epas1 genes in 10T1/2 cells. [file 12929_2021_770_MOESM2_ESM.pdf]

Shih et al. Additional file 2

Supplementary Figures

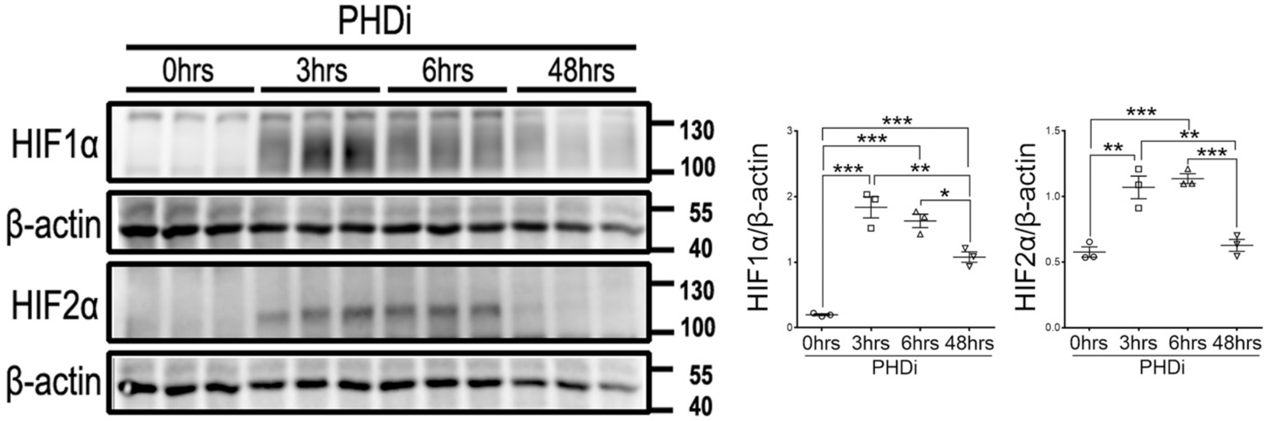

Figure S1. Prolyl hydroxylase domain inhibitor induces hypoxia-inducible factor 1α and hypoxia-inducible factor 2α in C3H10T1/2 cells. Representative Western blot analysis for the expression of hypoxia-inducible factor-1α (HIF1α) and HIF2α in the presence of prolyl hydroxylase domain (PHD) inhibitor (PHDi, roxadustat 50μM) for the indicated duration. Right panels show the expression of HIF1α and HIF2α normalized by β-actin. n = 3 per group. Data are expressed as the mean ± standard error of the mean (SEM). *P < 0.05, **P < 0.01, ***P < 0.001 by one-way ANOVA with Tukey's test.

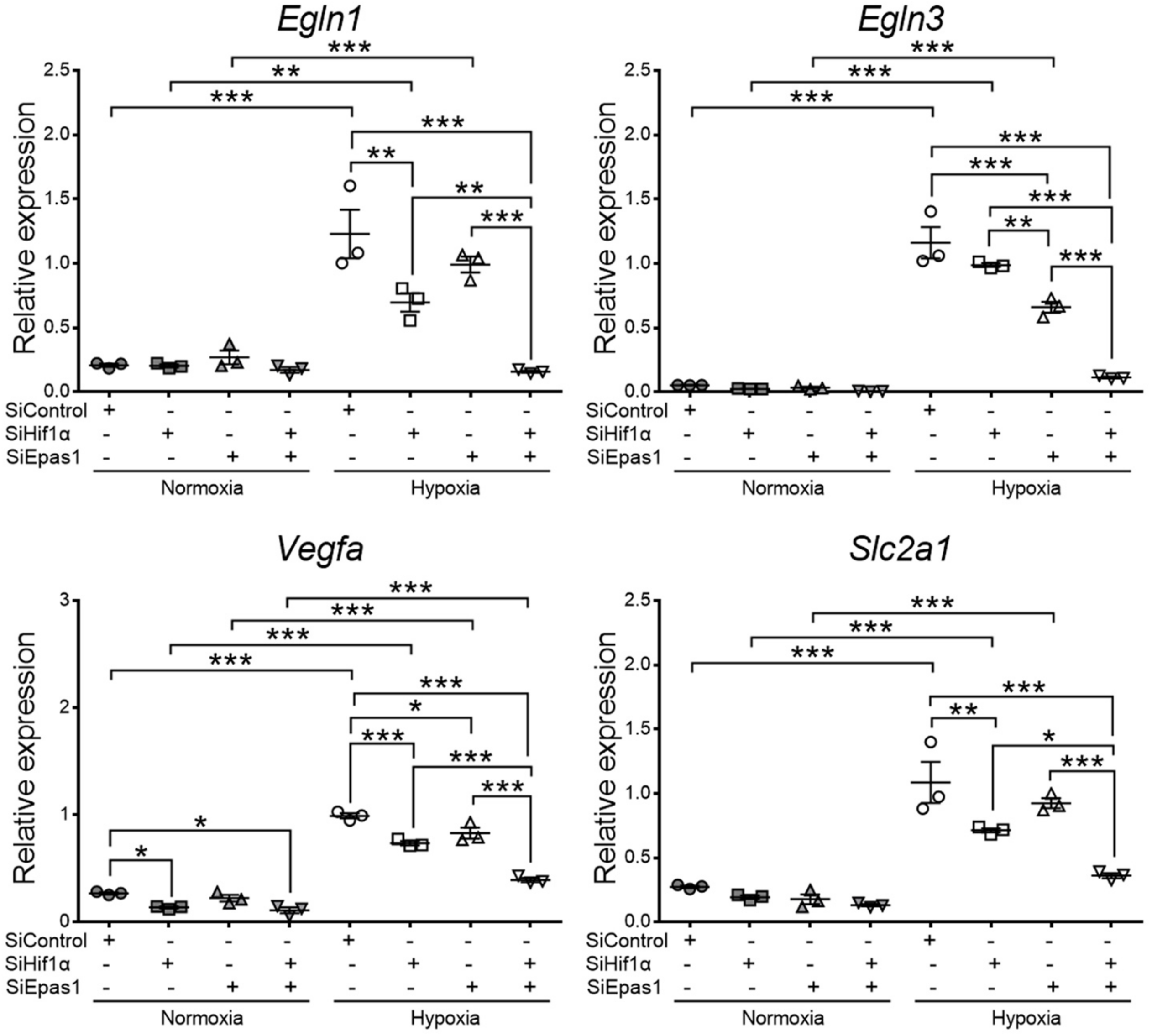

Figure S2. SiRNA transfection specific for *Hif1a* and *Epas1* downregulates hypoxia-induced genes. Schema illustrating the siRNA transfection specific for *Hif1a* (siHif1a), *Epas1* (siEpas1) or control (siControl), culture medium refresh, and gene analysis in Figure 3d. The relative mRNA expression of *Egln1, Egln3, Vegfa* and *Slc2a1* normalized by *Hprt*. *Hif1a*, *Epas1*, *Egln1*, *Egln3, Vegfa*, *Slc2a1* and *Hprt* encode HIF1α, HIF2α, PHD2, PHD3, vascular endothelial cell growth factor-A, solute carrier family 2 member 1 and hypoxanthine-guanine phosphoribosyl transferase, respectively. n = 3 per group. Data are expressed as the mean ± SEM. *$P < 0.05$, **$P < 0.01$, ***$P < 0.001$ by one-

way ANOVA with Tukey's test.

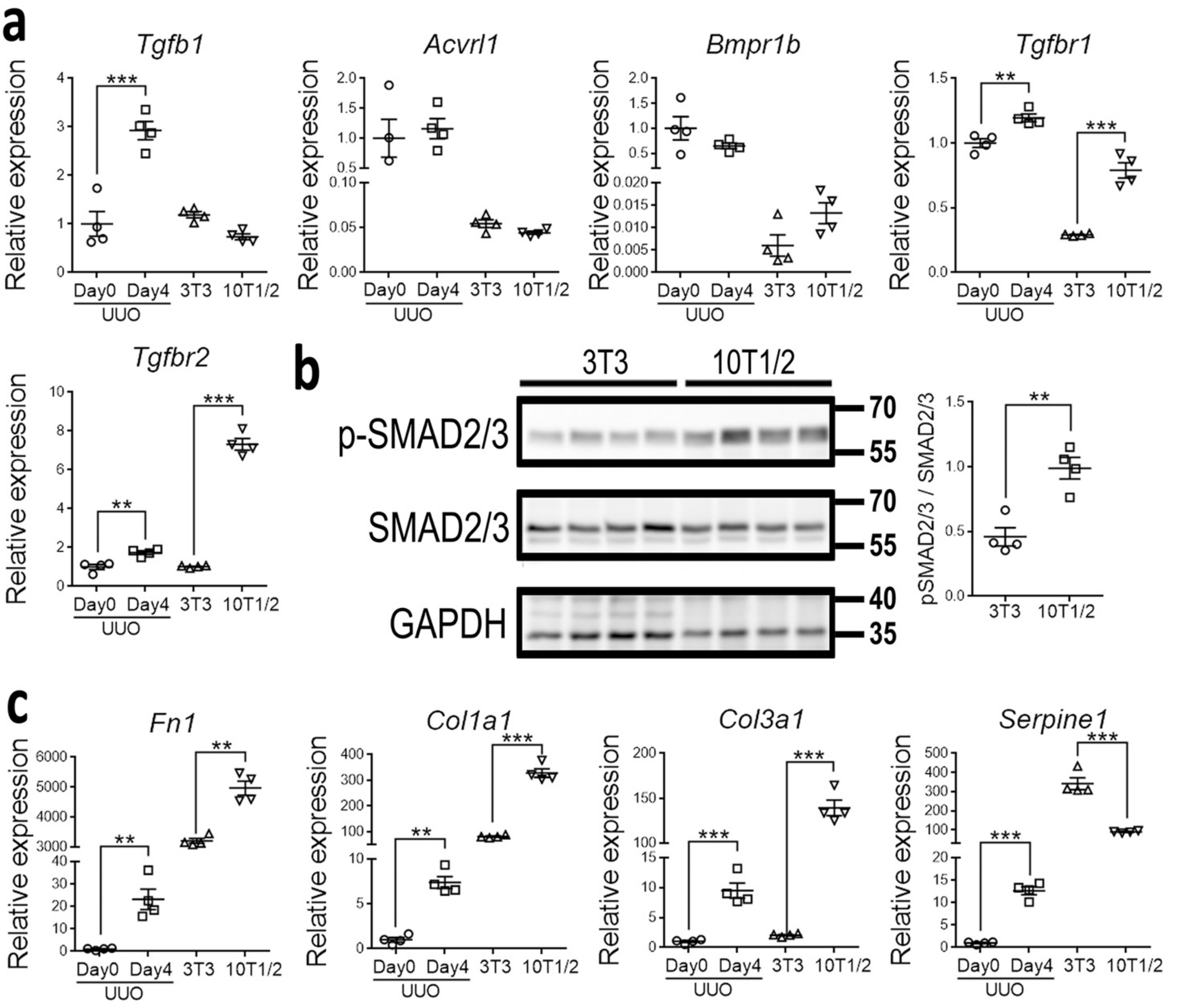

Figure S3. 10T1/2 cells exhibited higher levels of TGF-β1 signaling. (**a**) The relative mRNA expression of *Tgfb1*, *Acvrl1*, *Bmpr1b*, *Tgfbr1* and *Tgfbr2* normalized by *Hprt*. *Tgfb1*, *Acvrl1*, *Bmpr1b*, *Tgfbr1* and *Tgfbr2* encode TGF-β1, TGF-β superfamily receptors activin receptor-like kinase-1, bone morphogenetic protein receptor type 1B, TGF-β receptor 1 and TGF-β receptor 2, respectively. The mRNA expression of each gene in cells are compared to that of the kidney from day 0 (normal kidney) and day 4 after unilateral ureteral obstruction (UUO kidney) surgery. The relative mRNA expression of normal kidney is set at 1. (**b**) Representative Western blot analysis for phosphorylated SMAD2/3

(p-SMAD2/3), SMAD2/3 and GAPDH in 3T3 cells and 10T1/2 cells. Right panel showing the ratio of pSMAD2/3 / SMAD2/3 assessed by densitometry. (**c**) The relative mRNA expression of *Fn1*, *Col1a1, Col3a1* and *Serpine1* normalized by *Hprt*. *Fn1*, *Col1a1, Col3a1* and *Serpine1* encode fibronectin, type 1 collagen (α1 chain), type 3 collagen (α1 chain) and plasminogen activator inhibitor 1, respectively. Data are expressed as the mean ± SEM. **P < 0.01, ***P < 0.001 by Student's t test between cells and between kidneys.

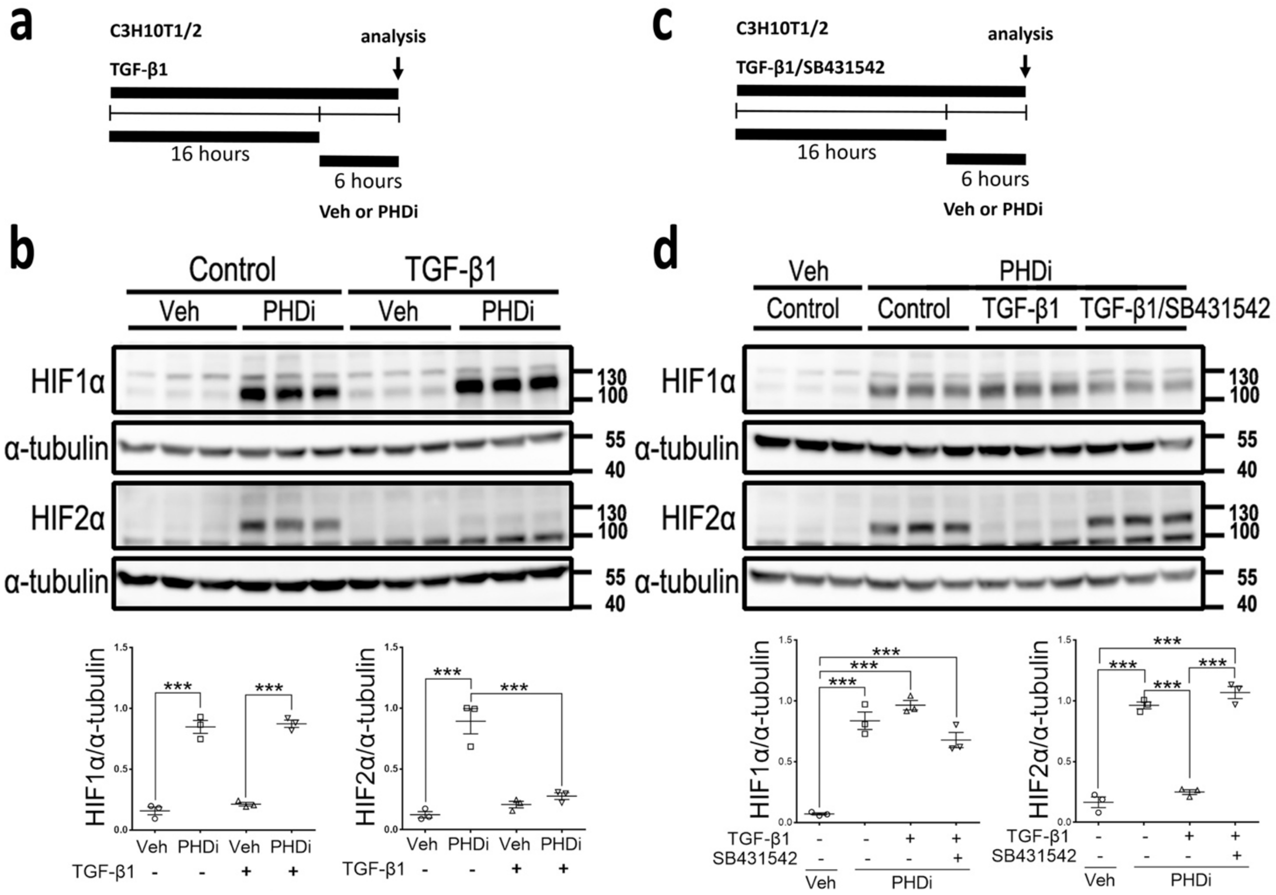

Figure S4. TGF-β1 inhibits PHDi-induced HIF2α expression through activin receptor-like kinase-5 in 10T1/2 cells. (**a**) Schema illustrating the analysis of HIFs for cells after 6-hour exposure to PHDi roxadustat or vehicle (Veh) in the presence or absence of TGF-β1 (5 ng/mL). (**b**) Representative Western blot analysis for HIF1α, HIF2α and α-tubulin in cells of the experiment in (a). Lower panels showing the expression of HIF1α and HIF2α normalized by α-tubulin. n = 3 per group. (**c**) Schema illustrating the analysis of HIFs for cells after 6-hour exposure to PHDi or Veh in the presence or absence of TGF-β1 (5 ng/mL) with or without activin receptor-like kinase-5 (ALK5) inhibitor (ALK5i) SB431542 (5 μg/mL). (**d**) Representative Western blot analysis for HIF1α, HIF2α and α-tubulin in cells of the experiment in (c). Lower panels showing the expression of HIF1α and

HIF2α normalized by α-tubulin. n = 3 per group. Data expressed as the mean ± SEM. ***$P < 0.001$ by one-way ANOVA with Tukey's test.

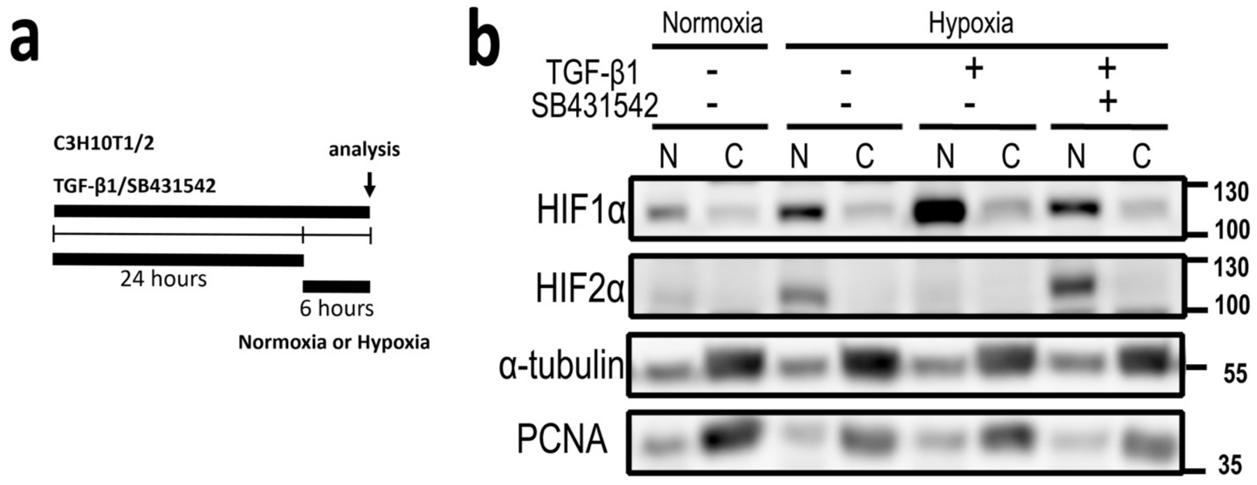

Figure S5. TGF-β1 inhibits hypoxia-induced nuclear accumulation of HIF2α through ALK5 in 10T1/2 cells. (**a**) Schema illustrating the analysis of HIFs for cells after 6-hour exposure to normoxia or hypoxia in the presence or absence of TGF-β1 with or without ALK5i SB431542. (**b**) Representative western blot analysis for the expression of HIF1α, HIF2α, α-tubulin and proliferating cell nuclear antigen (PCNA) in nuclear (N) or cytoplasmic (C) fraction.

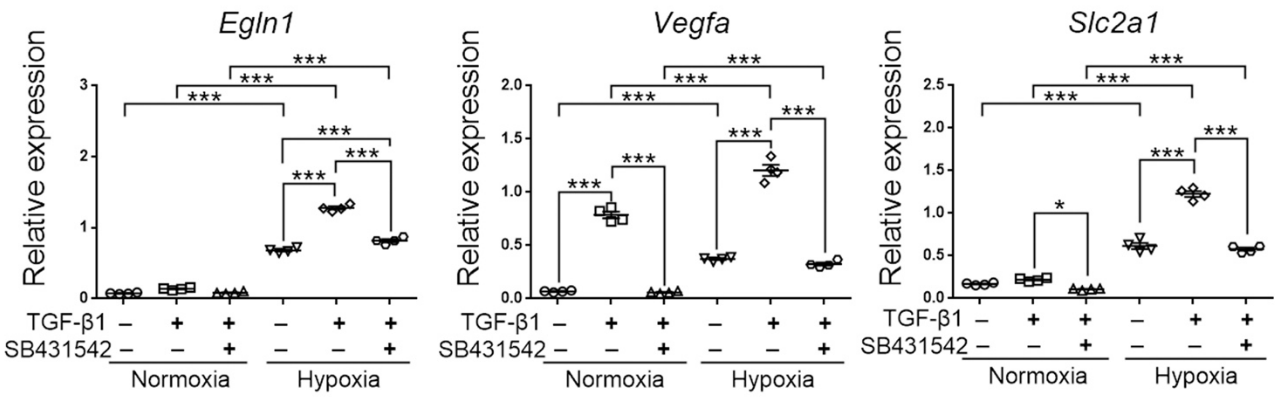

Figure S6. TGF-β1 increases hypoxia-induced expression of *Egln1*, *Vegfa* and *Slc2a1* through ALK5 in 10T1/2 cells. The relative mRNA expression of *Egln1*, *Vegfa* and *Slc2a1* normalized by *Hprt* in cells of the experiment in Figure 7f. n = 4 per group. Data are expressed as the mean ± SEM. *P < 0.05, ***P < 0.001 by one-way ANOVA with Tukey's test.

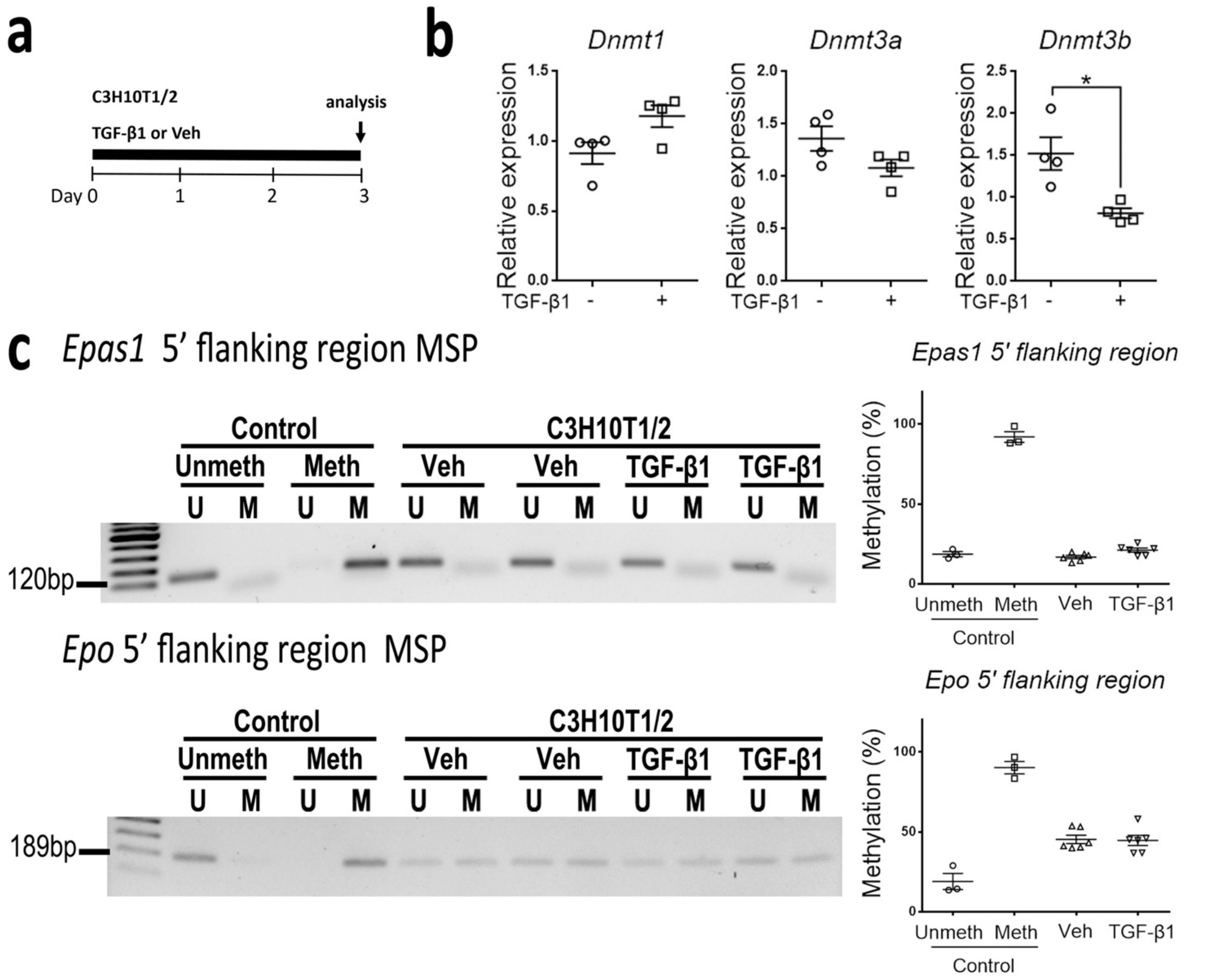

Figure S7. TGF-β1 does not change methylation in 5' flanking regions of *Epo* and *Epas1* genes in 10T1/2 cells. (**a**) Schema illustrating the analysis of 10T1/2 cells after 72-hour exposure to TGF-β1 (5 ng/mL) or Veh. (**b**) The relative mRNA expression normalized by *Hprt*. n = 4 per group. *Dnmt1*, *Dnmt3a* and *Dnmt3b* encode DNA methyltransferase 1 (DNMT1), DNMT3A and DNMT 3B, respectively. Data are expressed as the mean ± SEM. *$P < 0.05$ by Student's t test. (**c**) Representative electrophoresis of methylation specific PCR (MSP) using primers for the unmethylated (U) and methylated (M) 5' flanking regions of *Epas1* and *Epo* gene from 3 independent experiments. Right panels showing the percentage of 5' flanking region methylation determined by the densitometric

analysis of the MSP products. Data are expressed as the mean ± SEM. Meth, methylated; Unmeth, unmethylated controls.
